# An improved de novo assembling and polishing of *Solea senegalensis* transcriptome shed light on retinoic acid signalling in larvae

**DOI:** 10.1038/s41598-020-77201-z

**Published:** 2020-11-26

**Authors:** José Córdoba-Caballero, Pedro Seoane, Fernando M. Jabato, James R. Perkins, Manuel Manchado, M. Gonzalo Claros

**Affiliations:** 1 Department of Molecular Biology and Biochemistry, Universidad de Málaga, Málaga, 29071 Spain; 2 CIBER de Enfermedades Raras (CIBERER), Málaga, 29071 Spain; 3 Institute of Biomedical Research in Malaga (IBIMA), IBIMA-RARE, Málaga, 29010 Spain; 4 Consejería de Agricultura, Ganadería, Pesca y Desarrollo Sostenible, IFAPA Centro El Toruño, El Puerto de Santa María, Cádiz 11500 Spain; 5 Instituto de Hortofruticultura Subtropical y Mediterránea (IHSM-UMA-CSIC), Málaga, 29010 Spain

**Keywords:** Computational biology and bioinformatics, Cellular signalling networks, Genome informatics, High-throughput screening, Quality control, Animal breeding, Functional genomics, Genome, Sequencing, Transcriptomics

## Abstract

Senegalese sole is an economically important flatfish species in aquaculture and an attractive model to decipher the molecular mechanisms governing the severe transformations occurring during metamorphosis, where retinoic acid seems to play a key role in tissue remodeling. In this study, a robust sole transcriptome was envisaged by reducing the number of assembled libraries (27 out of 111 available), fine-tuning a new automated and reproducible set of workflows for de novo assembling based on several assemblers, and removing low confidence transcripts after mapping onto a sole female genome draft. From a total of 96 resulting assemblies, two “raw” transcriptomes, one containing only Illumina reads and another with Illumina and GS-FLX reads, were selected to provide SOLSEv5.0, the most informative transcriptome with low redundancy and devoid of most single-exon transcripts. It included both Illumina and GS-FLX reads and consisted of 51,348 transcripts of which 22,684 code for 17,429 different proteins described in databases, where 9527 were predicted as complete proteins. SOLSEv5.0 was used as reference for the study of retinoic acid (RA) signalling in sole larvae using drug treatments (DEAB, a RA synthesis blocker, and TTNPB, a RA-receptor agonist) for 24 and 48 h. Differential expression and functional interpretation were facilitated by an updated version of *DEGenes Hunter*. Acute exposure of both drugs triggered an intense, specific and transient response at 24 h but with hardly observable differences after 48 h at least in the DEAB treatments. Activation of RA signalling by TTNPB specifically increased the expression of genes in pathways related to RA degradation, retinol storage, carotenoid metabolism, homeostatic response and visual cycle, and also modified the expression of transcripts related to morphogenesis and collagen fibril organisation. In contrast, DEAB mainly decreased genes related to retinal production, impairing phototransduction signalling in the retina. A total of 755 transcripts mainly related to lipid metabolism, lipid transport and lipid homeostasis were altered in response to both treatments, indicating non-specific drug responses associated with intestinal absorption. These results indicate that a new assembling and transcript sieving were both necessary to provide a reliable transcriptome to identify the many aspects of RA action during sole development that are of relevance for sole aquaculture.

## Introduction

Flatfish refers to a diverse group of high valuable fish species worldwide belonging to the order Pleuronectiformes. Some of these species have been intensively studied in recent years due to their potential to be produced in aquaculture or as attractive biological models to understand the molecular mechanisms that govern the severe morphological and physiological transformations that occur during metamorphosis. In spite of the significant advances in the aquaculture of some species, most of them have to face new challenges related to growth performance, larval production, fish quality and pigmentation and disease resistance. As a result, abundant genomic resources including genomes and transcriptomes were reported and have been successfully used to understand flatfish asymmetry in Japanese flounder^1^, metamorphosis regulatory pathways in Atlantic halibut^2^, sex chromosome evolution and sex determination in half-smooth tongue sole^3^ and gene mapping in turbot^4^. It fact, fish genomics has been called to support genetic improvement of production traits and breeding programmes in aquaculture^5^.

*Solea senegalensis* (Senegalese sole) is the most important flatfish cultivated in Southern Europe by market price. Although its culture has faced specific challenges and some bottlenecks still persist, such as the lack of reproduction success for males cultured in captivity, the high dispersion of sizes or the high incidence of malformations. Nevertheless, more than 1600 tonnes are currently produced in Europe under advanced recirculation systems^6^. Key advances in hatchery and nursery protocols occurred in parallel with the development of important genomic resources including transcriptomes^7^ and genome drafts^8,9^. All this information enabled the design of useful mid- and high-density arrays and successful high-throughput transcript sequencing (RNA-seq) studies providing relevant information about lipid physiology in larvae^10^, olfactory communication between breeders^11^, immune response associated to experimental diets^12^, osmoregulation^7^ or pigmentation disorders in post-larvae^13^. However, the complex regulatory pathways that regulate metamorphosis in sole are not yet fully understood. The impressive plasticity of the individuals during this period is evidenced by their ability to imprint new properties as a function of the environmental factors. In fact, behavioral and metabolic changes lead to the acquisition of a functional digestive tract, competent immunity responses, mature neuro-muscular skeletal system, skin and pigmentation development, visual performance, and gonad development during this period^10,14–16^. Metamorphosis is triggered by a surge of thyroid hormones (TH)^17^ that govern a TH-responsive asymmetric centre in the anterior head region for eye migration and head remodeling^18^. Even so, these signalling cascades are highly modulated by environmental factors such as vitamin A that can interact with TH levels to modify skeletal morphogenesis leading to skeletal deformities^19^. In the Japanese flounder, retinoic acid (RA), the active metabolite of vitamin A, also appears to be critical in establishing asymmetric pigmentation and in modulating eye migration^1^. In sole, a few key genes involved in RA metabolism have been described^20^, and further research is required to understand the action of retinoids during metamorphosis in this species.

High-quality assembled and annotated transcriptomes are essential to achieve a comprehensive picture of cell or tissue physiology^21^, which is especially critical when genome sequence is not established. Transcriptomes are highly dynamic and show important differences depending on the cell genetic background, regulatory programming during organism development and cell differentiation, and post-transcriptional modifications^22^. As a result, assembling a de novo transcriptome, an essential approach for high-throughput gene expression studies, is prone to overestimate the number of transcripts due to immature mRNAs, intermediary spliced forms, sequencing errors, fragmented transcripts, insufficient sequencing depth and biological variability^23^. Previous assembled transcriptomes in sole reported a high number of transcripts^7,8,24,25^ that clearly exceeded the expected number of predicted genes as reported in closely related flatfish (about 21,000 protein-coding genes^1,3,26^). Accurate transcript quantification for gene expression studies is hindered by over-represented transcriptomes^27^, resulting in biased transcript discovery, over-estimation of family-collapsed contigs, and under-estimation of redundant contigs^11,27^. Bioinformatic strategies to reduce artefacts and redundancy have been implemented in sole^7^, but the result was still far from being optimal, and further polishing to increase tissue representativity, transcript completeness and annotations is required.

The release of better de novo transcriptomes is challenging that evolves quite fast^22,28,29^. The current study describes a new and improved version of the *S. senegalensis* transcriptome named SOLSEv5.0 using only a representative selection of sequencing libraries as input to a fine-tuned de novo assembling workflow based on *TransFlow*^28^ that combines multiple assemblers. The best assembly was then polished to remove low confidence transcripts. Finally, its suitability for RNA-seq studies was demonstrated using an experimental study that investigates the role of RA on metamorphic larvae by using a specific RA-receptor agonist or a RA synthesis blocker. The bioinformatic analysis was performed using an improved version of *DEGenes Hunter*^30^.

## Results

### De novo assembling for new “raw” transcriptomes

It is well established that more input reads do not produce better assemblies^31,32^. According to this observation, 30 Illumina and 1 GS-FLX libraries were adopted as the most informative ones from the original dataset of 111 libraries^7^ following criteria detailed in “Methods” section. These libraries accounted for 454 million Illumina pair-end reads and 5.66 millions GS-FLX single-end reads that after pre-processing rendered 361 and 3.1 millions of useful reads, respectively (Supplementary File 1). To validate species-specific reads, useful reads were then mapped onto a draft genome of *S. senegalensis*^8,9,33^. Supplementary File 1 shows that GS-FLX reads presented the lowest mapping rate (10.59 %, maybe due to their long length or the presence of too many polymorphisms), while Illumina libraries H634, H638 and H639 have distinctly higher mapping rates ($>70$ % maybe due to an overrepresentation of immature transcripts containing introns). Since libraries H634, H638 and H639, together with H632, belonged to the same experimental batch, they were discarded, resulting in a subset of 26 Illumina (275,501,704 paired-end reads) and 1 GS-FLX libraries for assembling the new transcriptome.

“Raw” transcriptomes were assembled using a fine-tuned *TransFlow* workflow detailed in “Methods” section. Since transcriptome evaluation was independent of the TT sequence but based on 15 quality parameters calculated by *TransFlow*, zebrafish transcriptome was selected as reference because it is the best known fish transcriptome. The 15 assemblies with the lowest PCA distance to the zebrafish reference transcriptome are presented in Table 1. scOases_k45 (Table 1), where only Illumina reads were assembled using *Oases* with *k*-mer = 45, was the first choice, and its name was simplified for convenience as “Oases_k45” from now on. Since GS-FLX libraries were prepared using a different set of tissues^7^, aaMin2/scOases_cat_cd/454Cap3 in Table 1 (named as “Min2_Oases_Cap3” from now on for convenience) was also considered. It was built using the following steps: (i) assembling of GS-FLX reads using *Mira3* and *Euler-SR*, and the resulting contigs were combined with *CAP3*; (ii) assembling Illumina reads with *Oases* using *k*-mers of 45 and 55, and redundancy decreasing using *CDHIT-EST*; and (iii) reconciliation of non-redundant contigs from step (ii) with *CAP3* contigs from step (i) using *Minimus2*. Interestingly, the Oases_k45 was produced in step (ii), which makes it a part of Min2_Oases_Cap3.

**Table 1:** The 15 assembling approaches with the lowest PCA distance to the reference transcriptome of *D. rerio* (zebrafish) generated after fine-tunning *TransFlow* are presented.

Assembling name^a^	PCA distance	Platforms
scOasesK45*	0.0052	Illumina
scOasesK55	0.0141	Illumina
scOases_cat_cd	0.0162	Illumina
ctRay_cat_cd_rcMin2	0.0423	Illumina
scOases_cat_cd_rcMin2	0.0562	Illumina
ctRay_cat_cd	0.0577	Illumina
ctRayK45	0.0721	Illumina
ctRayK55	0.1056	Illumina
aaMin2/scOases_cat_cd/454Cap3*	0.1125	Illumina + GS-FLX
aaMin2/scOasesK45/454Cap3	0.1195	Illumina + GS-FLX
aaMin2/scOases_cat_cd_rcMin2/454Cap3	0.1201	Illumina + GS-FLX
aaMin2/scOasesK55/454Cap3	0.1297	Illumina + GS-FLX
aaMin2/ctRay_cat_cd/454Cap3	0.1584	Illumina + GS-FLX
aaMin2/ctRay_cat_cd_rcMin2/454Cap3	0.1614	Illumina + GS-FLX
aaMin2/ctRayK45/454Cap3	0.1759	Illumina + GS-FLX

The 8 top-ranked ones were based only on Illumina libraries and the remaining were combinations of Illumina and GS-FLX reads. “Raw” transcriptomes selected for further investigation are marked with “*”.

^a^Names are derived from the resulting *TransFlow* approach as described in^28^.

Both Oases_k45 and Min2_Oases_Cap3 were functionally and structurally annotated with full-length proteins from *Actinopterigii* taxon and then completed with full-length proteins from the vertebrate division, and were then compared to the published transcriptome (referred as v4.0)^7^. Table 2 shows that new “raw” transcriptomes have less tentative transcripts (TTs), smaller overall transcriptome length (but similar between Oases_k45 and Min2_Oases_Cap3), a two- to four-fold increase of N50 and N90, and less artefacts than v4.0 obtained with the same reads. Therefore, the proposed fine-tuned assembling workflow generated more accurate transcriptomes.

**Table 2:** Main features of “raw” transcriptomes (the original v4.0 as well the new Min2_Oases_Cap3 and Oases_k45) after functional and structural annotation.

Feature	v4.0	Min2_Oases_Cap3	Oases_k45
Transcripts	697,125	153,847	87,362
Aggregate transcriptome length (nt)	366,337,327	147,880,071	142,273,313
Longest transcript (nt)	40,163	41,091	18,479
Indeterminations (%)	0.45	0.67	0.44
**Artefacts**	125,606	841	90
Misassembled	300	123	10
Unmapped	125,306	718	80
N50 (nt)	1292	2051	2659
N90 (nt)	180	360	825
Tentative transcripts (%)	81.98	99.45	87.87
**With orthologue**	69,525	31,110	23,757
Unique orthologue IDs	37,607	22,948	14,374
Complete transcripts	27,303	14,710	15,034
Unique complete transcripts	15,402	11,219	7673
Putative ncRNA	9811	3756	2500
**Without orthologue**	492,182	118,140	50,515
Coding sequence	57,619	24,999	17,529
Unknown nature	434,563	93,141	32,986

The second half of Table 2 displays the functional annotation and coding statuses of the three transcriptomes. It was striking that the new “raw” transcriptomes had less TTs ‘Without orthologue’ and without predictable coding region (‘Unknown nature’ in Table 2) than v4.0. Furthermore, Min2_Oases_Cap3 had 7 353 additional orthologues (‘With orthologue’ in Table 2), 8574 additional unique othologues and 3546 additional unique complete transcripts compared to Oases_k45. Therefore, Min2_Oases_Cap3 appeared as a more comprehensive “raw” transcriptome than Oases_k45 despite having a high rate of unknown TTs, which indicated that further polishing was advisable.

### Transcriptome polishing

The first cleansing step was conducted by mapping the TTs from the three “raw” transcriptomes (see Table 2) on the Senegalese sole genome, where v4.0 was included as a baseline control. As a result, Min2_Oases_Cap3 and Oases_k45 transcriptomes contained less unmapping, atypical and low quality TTs than v4.0 (see Table 3 for values and definitions). Hence, the three kinds of low confidence TTs were removed from their respective “raw” transcriptome to produce “definitive” transcriptomes that were then evaluated for intron-exon patterns. A higher proportion of introns per TT were found in Min2_Oases_Cap3 and Oases_k45, particularly for TTs with $> 3$ exons (Table 3). Most removed TTs were very short, or the consequence of chimaeric or misassembled contigs (Supplementary File 2). Therefore, polishing steps improved “definitive” transcriptomes, even though it hardly removes low confidence TTs in v4.0 due to a suboptimal assembling approach.

**Table 3:** Low confidence TTs identified in “raw” transcriptomes that were removed to constitute “definitive” transcriptomes whose TTs were counted by the number of exons identified in their sequence.

Transcriptome name	“Raw” transcriptomes			“Definitive” transcriptomes					
	$\bf Unmapping^{a}$	$\bf Atypical^{b}$	Low quality^c^	Gapped by 1 to $>5$ exons (%)					
	(%)	(%)	(Amount)	1	2	3	4	5	>5
v4.0	8.95	73.21	5,040	0.25	4.87	2.36	1.70	1.31	6.63
Oases_k45	1.89	52.80	3,694	0.78	0.37	4.61	3.99	3.59	27.74
Min2_Oases_Cap3	5.21	58.44	4,543	0.32	7.70	4.67	3.44	2.75	14.52

^a^‘Unmapping’ refers to TTs that were not mapped onto the genome draft of Senegalesse sole, suggesting that they come from artefactual assembling.

^b^‘Atypical’ refers to TTs that were not gapped by introns after mapping onto the genome draft, suggesting that they are closer to a genomic sequence than to a transcript.

^c^‘Low quality’ refers to TTs that mapped $<70$% of their length with $<70$% identity onto the genome draft, suggesting that their sequences are chimaeric or contain too many errors.

**Figure 1:**
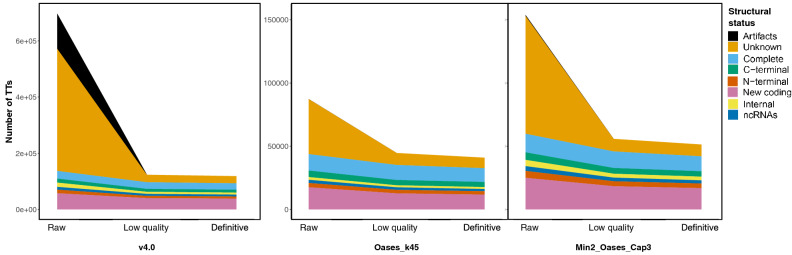
Distribution of TTs grouped by their structural status in the three assembling approaches: v4.0, Oases_k45 and Min2_Oases_Cap3. “Raw” refers to transcriptomes as in Table 2. “Low quality” results from removal of unmapped and atypical TTs in Table 3. “Definitive” are also devoid of low quality TTs in Table 3. Structural statuses for TTs are ‘Artifacts’ (not supported by mapped paired-end reads), ‘Unknown’ (without any sequence similarity in databases), ‘Complete’ (with a complete CDS (coding sequence) region), ‘C-terminal’ (with a C-terminal part of a CDS), ‘N-terminal’ (with a N-terminal part of a CDS), ‘Internal’ (internal CDS identified, lacking both N- and C-terminus), ‘New coding’ (can code for an unidentified protein), and ‘ncRNA’ (identical to ncRNA precursor in databases).

To confirm that polishing did not remove informative TTs, structural statuses of “raw” and “definitive” transcriptomes, as well as the set of “raw” quality” TTs, were compared (Fig. 1). ‘Unknown’ status was always the most abundant and accounted for huge TT numbers in “raw” transcriptomes and was markedly decreased after polishing (Table 3). Artefacts—abundant in “raw” v4.0 (125,606)—were nearly absent in Min2_Oases_Cap3 and Oases_k45 (841 and 90, respectively), and most of them (99.17%: 25,939 not mapping onto the genome draft and 98,625 considered atypical) were removed after polishing. Therefore, any TT tagged with ‘Artifact’ status was removed. All of this produced a consistent number of genes between Min2_Oases_Cap3 and Oases_k45 (51,348 and 40,950, respectively) compared to the inflated number of v4.0 (118,436 TTs). Hence, the results supported that polishing removed TTs of limited interest.

**Figure 2:**
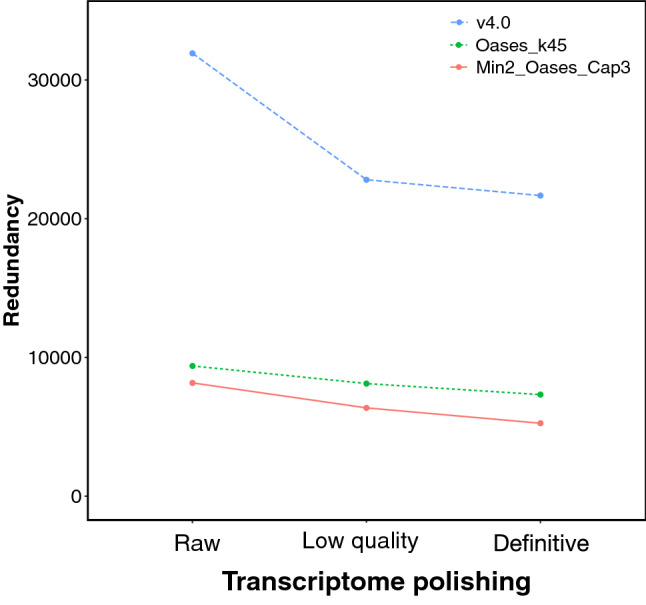
Transcriptome polishing reduces redundancy. Values were computed as the difference between the amount of TTs with orthologues and “Unique orthologue IDs” (Table 2) for each polished transcriptome as in Fig. 1.

Intriguingly, the decrease in the other structural statuses after polishing was moderated (Fig. 1). This was explained by the TT redundancy (Fig. 2), where v4.0 was the most redundant compared to Oases_k45 and Min2_Oases_Cap3. TT decreases from “low quality” to “definitive” transcriptomes may be explained by redundancy too. Overall, these data demonstrate that “definitive” Min2_Oases_Cap3 was less redundant and contained more different orthologue IDs than the other transcriptomes and hence it should be selected as SOLSEv5.0.

**Figure 3:**
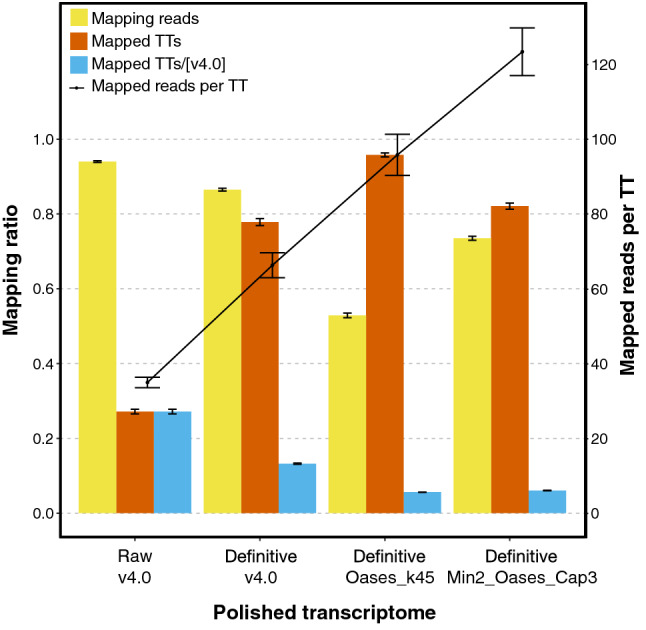
Mapping ratios of the 18 libraries described in Supplementary File 3 when “definitive” transcriptomes of v4.0, Oases_k45 and Min2_Oases_Cap3, as well as the “raw” v4.0 transcriptome as a baseline control, were used as reference. Ratios were calculated as the amount of sequences that align to a transcriptome with respect to the number of TTs in the transcriptome and are given as mean and standard error for the 18 samples. “Mapping reads” refers to reads aligned to the corresponding reference transcriptome; “Mapped TTs” refers to TTs that contain at least one read-pair aligned on them; “Mapped TT/[v4.0]” is as “Mapped TTs” but divided by the amount of TTs in the “raw” v4.0; and “Mapped reads per TT” refers to the amount of mapped reads on the transcriptome per total number of TTs.

### The SOLSEv5.0 transcriptome

Usefulness of SOLSEv5.0 transcriptome for gene expression analyses was demonstrated by mapping 18 Illumina libraries corresponding to a study of RA signalling in Senegalese sole larvae (Supplementary File 3) onto the three “definitive” transcriptomes and the “raw” v4.0 as a baseline control (Fig. 3). Columns “Mapping reads” and “Mapping TTs/[v4.0]” indicated that the amount of reads mapping on a transcriptome decreased with respect to the number of TTs in the transcriptome. The ratio of mapping reads in “definitive” Oases_k45 was lower (50 %) than in Min2_Oases_Cap3 (75 %), that could be due to the tissue-specific TTs provided by the GS-FLX reads, suggesting that they should be retained in SOLSEv5.0. The fact that most of the TTs ($>80$ %) in “definitive” Min2_Oases_Cap3 and Oases_k45 were supported by at least one pair of reads (“Mapped TTs” columns in Fig. 3) reinforced again that polishing was not associated with information loss but a redundancy reduction, as suggested above (Fig. 2). Finally, “definitive” Min2_Oases_Cap3 showed the highest amount of “Reads per TT” (Fig. 3), strongly suggesting that it should be the Senegalese sole transcriptome SOLSEv5.0^34^.

When SOLSEv5.0^34^ and “definitive” Oases_k45 (Table 4) were compared with their “raw” transcriptome versions (Table 2, Fig. 1), it is noticeable the strong reduction of TTs without orthologue and, more interestingly, that most of them were now predicted as coding sequences by *Transdecoder* (which is included in our *Full-LengtherNext* annotation tool) instead of unknown nature. It is also observed an increase in N50 and N90 of each assembly, and a decrease in artefacts, in spite of the decrease in the number of orthologues and unique complete transcripts due to polishing (even if this reduction is mainly due to redundancy, as observed in Fig. 2). The main features of SOLSEv5.0 when compared to “definitive” Oases_k45 (Table 4) revealed that (i) the aggregate length of SOLSEv5.0 was shorter in spite of having more TTs; (ii) artefactual TTs (Supplementary File 2, Fig. 1) were absent; (iii) SOLSEv5.0 had more orthologues, more unique IDs and more complete and unique complete TTs in spite of having shorter N50 and N90; and (iv) although SOLSEv5.0 had 6088 more TTs without orthologue, a higher proportion (5098, 64.2 %) was predicted to code for a protein that can be the result of new genes, genes with highly divergent sequences, or new splicing isoforms that should be taken into account. Note that the longest TT observed in Min2_Oases_Cap3 in Table 2 disappeared in Table 4, and both “definitive” transcriptomes have the same longest transcript. Since it is reported that the overall quality of assemblies must rely on more than one statistics^35,36^, results about mapping of Fig. 3 as well as the increase of N50/N90, the contrast in number of orthologues, unique orthologues, complete transcripts, and even the ratio of coding sequences among the TTs without orthologue (Table 4) are also reinforcing the choice of the SOLSEv5.0 transcriptome.

**Table 4:** Main features of “definitive” transcriptomes based on Min2_Oases_Cap3 (SOLSEv5.0) and Oases_k45.

Feature	SOLSEv5.0	Oases_k45
Tentative transcripts	51,348	40,950
Aggregate length (nt)	91,602,755	98,021,925
Longest TT (nt)	23,792	23,792
Indeterminations (%)	0.103	0.002
Artefacts	0	0
N50 (nt)	2836	3182
N90 (nt)	822	1328
**With orthologue**	22,684	19,113
Unique orthologue IDs	17,429	11,795
Complete transcripts	11,798	10,883
Unique complete transcripts	9527	6591
Putative ncRNA	2494	1755
**Without orthologue**	26,170	20,082
Coding sequence	16,808	11,710
Unknown nature	9362	8372

### Differential expression of RA signalling in sole larvae

To obtain insights about RA-mediated regulatory cascades in sole metamorphosing larvae, counts of mapped reads of Supplementary File 3 were analysed using a new version of *DEGenes Hunter*^30^. It was designed for dealing with de novo assembled transcriptomes with improved functionalities for differential expression and new capabilities for functional interpretation. A highly conservative approach applying default expression thresholds based on “prevalent” DETs (differentially expressed TTs) was followed. Untreated control (CTRL) larvae were compared to those conditioned with DEAB (a blocker of RA synthesis that inhibits the enzyme retinaldehyde dehydrogenase) and TTNPB (a specific RA-receptor agonist that triggers a RA-signalling response) at 24 and 48 h, resulting in four sets of DETs (folders starting by CTRL_vs in Supplementary File 4).

**Figure 4:**
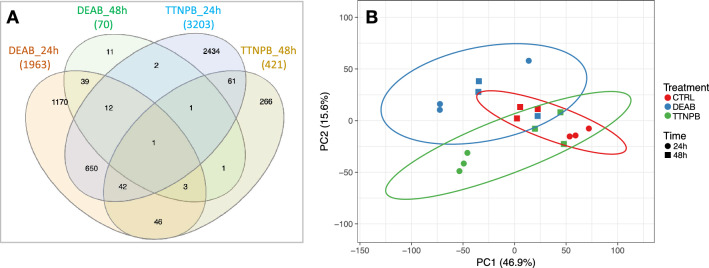
Distribution of DETs in Supplementary File 4 by time and treatment. (**A**) Venn diagram representing the number of DETs by DEAB and TTNPB treatments and by time (24 and 48 h) with respect to CTRL (Control). Total amounts of DETs are indicated in parentheses. (**B**) Principal component analysis (PCA) plot using DETs. It shows three distinct clusters (enclosed by ellipses) that indicate CTRL (red), DEAB (blue) and TTNPB (green) treatments. Sampling points are identified with circles (24 h) and squares (48 h).

A total of 1963 and 70 DETs were identified in DEAB-treated larvae and 3203 and 421 in the TTNPB-treated larvae with respect to CTRL at 24 and 48 h after treatment, respectively (Fig. 4A). When DETs were compared between sampling points within each treatment, 79 % matched between 24 h and 48 h in DEAB while only 25 % coincided in TTNPB-treated larvae at 24 and 48 h (Fig. 4A). Interestingly, both drug treatments modified the expression of a common set of 755 transcripts (mainly at 24 h, 650 DETs). The PCA (principal component analysis) using the 4 730 “prevalent” DETs across the four comparisons displayed a transient effect more intense at 24 h than at 48 h. The three experimental groups (CTRL, DEAB and TTNPB) showed separation at 24 h (Fig. 4B, circles). However, at 48 h (Fig. 4B, squares) the DEAB-treated larvae appeared closer and overlapping with the CTRL group although TTNPB still remained slightly separated, suggesting a more sustained response with TTNPB than with DEAB.

To assess the robustness of SOLSEv5.0 regarding differential expression, the same analysis was performed using “raw” v4.0 as transcriptome reference and the results are given Supplementary File 5. In agreement with Fig. 3, the average number of normalized counts per TT for SOLSEv5.0, ranging from 40 % (48 h) to 90 % (24 h), is higher than for “raw” v4.0 (Supplementary File 6A). As expected, more DETs were identified in “raw” v4.0 (Supplementary File 6B) due to redundancy (Fig. 2) and the total number of TTs (697 125 for “raw” v4.0 (Table 2) and 51 348 for SOLSEv5.0 [Table 4]). The mapping of 48.4 % of TTs in “raw” v4.0 produce more common DETs than using SOLSEv5.0 (compare Supplementary File 6B with Fig. 4A). However, sample clustering by PCA in Supplementary File 6C was similar to Fig. 4B. Taken together, it can be stated that the highly polished SOLSEv5.0 was less redundant, less confounding and at least as informative than the original “raw” v4.0 transcriptome.

### Functional analysis of DEAB and TTNPB effects on RA signalling

**Figure 5:**
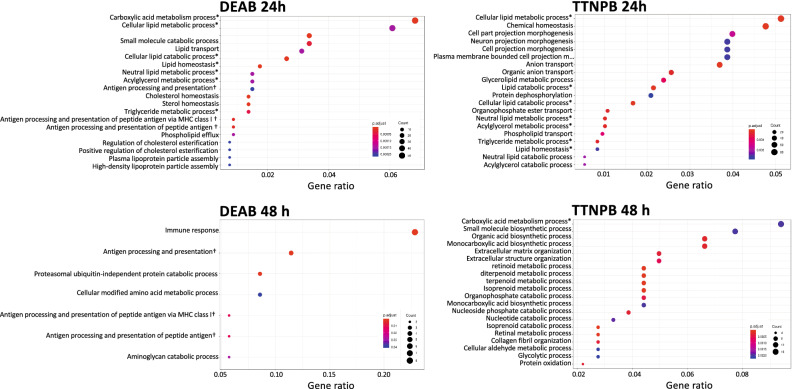
Top 20 significant GOs obtained from the enrichment analysis of DETs identified in the DEAB and TTNPB treatments after 24 and 48 h using SOLSEv5.0. Categories were sorted by gene ratio (proportion of DETs present in the total amount of genes assigned to a GO category), represented by the X axis. Point sizes reflect the number of DETs on each category. Colours represent the adjusted *P*-value of each category. *Categories represented both in DEAB and TTNPB treatments. ${^{\dag}}$Categories represented at both times within the same treatment.

The new version of *DEGenes Hunter* extended and facilitated the functional data interpretation based on zebrafish orthologues (given as functional_report.html in every CTRL_vs folder in Supplementary File 4). Overall, main GOs of biological pathways modified by drug treatments are depicted in Fig. 5. DEAB and TTNPB treatments shared eight over-represented pathways related to “lipid metabolism”, “transport and homeostasis” and “carboxylic acid metabolism”. Pathways more represented in DEAB-treated larvae were “regulation of cholesterol esterification” and those associated with immune system (“antigen processing and presentation”, “antigen processing and presentation of peptide antigen via MHC class I”, “antigen processing” and “presentation of peptide antigen”, the latter was observed both at 24 and 48 h). In TTNPB-treated larvae, specific over-represented pathways were mainly related to morphogenesis at 24 h and those related to retinoids and other metabolic pathways at 48 h.

**Figure 6:**
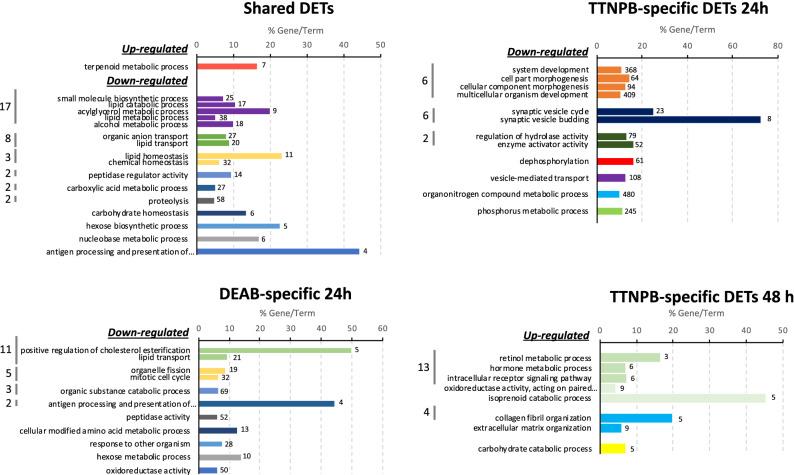
GO analysis using shared DETs between DEAB and TTNPB and those treatment-specific at 24 or 48 h (DEAB specific DETs at 48 h were not represented since 11 DETs are not enough for enrichment analysis). Bars represent significantly enriched terms in Biological Process category of GOs generated by *ClueGO*. Colours mark different interrelated GO terms. The number at the end of each bar indicates the number of DETs in that term.

To gain more insights about RA-signalling, the set of shared DETs and those specific for DEAB and TTNPB treatments were separately analyzed for enrichment of GO categories. In shared DETs, the “terpenoid metabolic process” pathway that is directly involved in RA metabolism was significantly over-represented and mainly up-regulated. Moreover, genes in categories previously identified and associated with “lipids”, “immune system”, “carbohydrate homeostasis” and “proteolysis” appeared mainly down-regulated (Fig. 6, ‘Shared DETs’). For DEAB-specific DETs (1220), main over-represented pathways contain down-regulated genes at 24 h (1023 DETs). Some of them were previously observed in the set of shared DETs (those related to “peptidase activity”, “immune system” and “lipids”) and only “mitotic cell cycle” and “organelle fission” pathways were specific (Fig. 6, ‘DEAB-specific 24h’). When the set of TTNPB-specific transcripts was analyzed at 24 h (2496 specific DETs, 83.5% down-regulated) main over-represented GO categories were related to “morphogenesis”, “phosphorus and organonitrogen metabolism”, “vesicle-mediated transport”, “dephosphorylation”, “enzyme activator activity” and “synaptic vesicle cycle” (Fig. 6, ‘TTNPB-specific 24h’). At 48 h (327 DETs, 84.7% up-regulated) there was a shift in significantly enriched categories with the “regulation of carbohydrate catabolism”, “extracellular matrix organization”, “isoprenoid catabolic process”, “intracellular receptor signalling pathway” and “retinol metabolic process”, among others, pathways (Fig. 6, ‘TTNPB-specific 48 h’).

The same functional interpretation was carried out using the results Supplementary File 5 obtained with “raw” v4.0 transcriptome as reference. In spite of the different number and DETs and zebrafish orthologues (Supplementary File 7, Venn diagrams), with about 60.4 % matching in zebrafish IDs in both sets of DETs), the GO analysis clearly demonstrated that categories specially related with RA metabolism of TTNPB at 48 h were missed (Supplementary File 8). In fact, “raw” v4.0 transcriptome was biased toward metabolic pathways with a high redundancy through the different comparisons. These categories were also observable using SOLSEv5.0 (Fig. 6), supporting that SOLSEv5.0 can provide a clearer and more comprehensive functional interpretation, avoiding the negative consequences of high redundancy.

**Table 5:** Representative DETs between DEAB and TTNPB with respect to CTRL at 24 and 48 h after RNA-seq analysis.

Tentative	${\hbox {Description}}^{a}$	Gene	DEAB		TTNPB	
transcript ID		name	24 h	48 h	24 h	48 h
**RA signalling**						
SOLSEv5.0_49442	Retinoic acid receptor $\gamma$ B	rargb	NA	NA	NA	1.73*
SOLSEv5.0_14451	Retinoic acid receptor $\alpha$ A	raraa	${-0.87}$	0.34	${-}1.81$*	0.88
SOLSEv5.0_08337	Retinoic acid receptor RXR-$\beta$-A	rxrba	${-}{1.11}$	${-}{0.31}$	${-}{1.46}$*	${-}{0.14}$
SOLSEv5.0_50356	Cellular retinoic acid binding protein 1	crabp1a	1.11*	0.01	1.28*	${-}{0.59}$
SOLSEv5.0_16248	Cellular retinoic acid-binding protein 2	crabp2b	${-0.30}$	0.05	1.33*	1.00
SOLSEv5.0_31100	Retinol binding protein 4	rbp4	${-1.27}$*	${-0.13}$	${-1.17}$*	0.05
SOLSEv5.0_14047	Diacylglycerol O-acyltransferase 1	dgat1a	${-1.42}$*	${-0.25}$	${-1.76}$*	0.34
SOLSEv5.0_12778	Aldehyde oxidase 1	aox1	${-1.30}$*	0.20	$-$1.05*	0.73
**RA biosynthesis and degradation**						
SOLSEv5.0_18848	Lecithin retinol acyltransferase	lrata	NA	NA	2.22*	5.32*
SOLSEv5.0_22413	Lecithin retinol acyltransferase-like	lratb.1	1.10	0.05	2.78*	3.58*
SOLSEv5.0_15374	Putative all-trans-retinol 13,14-reductase	retsat	$-$1.51	$-$0.65	$-$1.82*	$-$3.11
SOLSEv5.0_49456	Retinol dehydrogenase 7	rdh7	$-$1.68*	$-$0.25	$-$1.72*	0.48
SOLSEv5.0_07671	Dehydrogenase/reductase 3	dhrs3b	$-$2.57*	$-$1.97	1.35*	5.34*
SOLSEv5.0_32529	Aldehyde dehydrogenase 1 family member A2	aldh1a2	$-$1.07	0.75	$-$1.92*	1.21
SOLSEv5.0_28106	$\beta ,\beta$-carotene 15,15’-dioxygenase-like	bco1	$-$3.34*	$-$0.08	$-$1.17	0.15
SOLSEv5.0_48894	$\beta ,\beta$-carotene 9’,10’-oxygenase-like	bco2a	1.32*	0.12	0.57	$-$0.43
SOLSEv5.0_10122	$\beta ,\beta$-carotene 9’,10’-oxygenase-like	bco2l	$-$2.23*	$-$0.32	$-$1.07*	2.75*
SOLSEv5.0_12130	Cytochrome P450 26A1	cyp26a1	$-$0.91	$-$0.54	4.56*	7.75*
SOLSEv5.0_16663	Cytochrome P450 26B1	cyp26b1	$-$0.30	0.11	0.04	3.20*
**Thyroid axis**						
SOLSEv5.0_10022	Thyroglobulin	tg	$-$1.63	$-$0.43	$-$3.83*	0.17
SOLSEv5.0_11284	Thyroid hormone receptor $\alpha$-B	thrab	$-$0.74	$-$0.04	$-$1.71*	0.53
SOLSEv5.0_00904	Iodothyronine deiodinase 1	dio1	1.37*	0.91	0.62	$-$0.04
**Phototransduction signalling in retina**						
SOLSEv5.0_08034	Retinal-specific ATP-binding cassette transporter	abca4	2.25	$-$0.05	2.10*	0.05
SOLSEv5.0_03276	RPE-retinal G protein-coupled receptor	rgr	1.52*	1.41*	0.31	$-$0.42
SOLSEv5.0_07779	Retinal Mueller cells isomerohydrolase	rpe65b	0.74	$-$0.23	1.01*	1.13*
**Pigmentation**						
SOLSEv5.0_43495	GTP cyclohydrolase 1	gch1	$-$0.49	$-$0.71	0.44	1.28
SOLSEv5.0_16833	Purine nucleoside phosphorylase 5	pnp5a	$-$0.10	0.74	$-$1.32*	0.16
SOLSEv5.0_41612	Cytosolic purine 5’-nucleotidase	nt5c2	$-$0.77	$-$0.53	1.08*	1.88*
SOLSEv5.0_19961	G protein-coupled receptor 143	gpr143	1.12	NA	1.33	0.58
**Extracellular matrix**						
SOLSEv5.0_17376	Collagen $\alpha$-4(IV) chain	col4a4	0.76	0.07	1.08*	NA
SOLSEv5.0_41972	Collagen type IV $\alpha$ 5 chain	col4a5	$-$0.83	0.09	$-$1.64*	NA
SOLSEv5.0_10967	Collagen type IV $\alpha$ 6 chain	col4a6	$-$1.29	$-$0.02	$-$1.85*	NA
SOLSEv5.0_19543	Collagen $\alpha$-2(V) chain	col5a2a	$-$1.32	$-$0.06	$-$1.82*	NA
SOLSEv5.0_10008	Collagen type V $\alpha$ 3 chain	col5a3	$-$0.43	0.57	NA	1.27*
SOLSEv5.0_41833	Collagen type XI $\alpha$ 1 chain	col11a1b	$-$1.55*	0.20	$-$1.52*	NA
SOLSEv5.0_41831	Collagen type XI $\alpha$ 1 chain	col11a1a	$-$1.28*	$-$0.31	$-$1.20*	NA
SOLSEv5.0_14185	Collagen type XVI $\alpha$ 1 chain	col16a1	$-$1.48	$-$0.19	$-$1.04*	NA
SOLSEv5.0_20615	Collagen $\alpha$-1(XXI) chain	col21a1	$-$0.88	0.31	NA	1.29*
SOLSEv5.0_50025	Collagen $\alpha$-1(XXVIII) chain	col28a2a	$-$0.53	0.83	NA	2.11*
SOLSEv5.0_19261	Osteocalcin 2	bglap	$-$0.66	$-$0.89	NA	$-$2.20*
SOLSEv5.0_49404	Procollagen-lysine,2-oxoglutarate 5-dioxygenase 2	plod2	0.01	0.16	NA	1.19*
SOLSEv5.0_49653	Procollagen-lysine,2-oxoglutarate 5-dioxygenase 1	plod1a	$-$0.33	0.67	NA	1.98*

Transcript ID as in SOLSEv5.0, gene description, gene name, and mean logFC are indicated.

^a^DET descriptions confirmed by BLASTing their sequences at NCBI. *: Statistically significant fold-change. NA: logFC was not available for such comparison using the parameters established for the analysis with *DEgenes Hunter*.

A detailed analysis of DETs from Supplementary File 4 identified several genes related to RA and TH metabolism that are indicated in Table 5. RA receptors, such as *rargb, raraa* and *rxrba*, that mediated RA actions were differentially expressed only in the TTNPB-treated larvae. The *rbp4* (retinol binding protein) was down-regulated and *crpb1a* (cellular RA binding protein) was up-regulated in both DEAB and TTNPB treatments, whereas *crabp2b* was up-regulated only in TTNPB treatments. When enzymes implicated in RA biosynthesis and degradation were monitored, cytochromes P450 26A1 (*cyp26a1*) and 26B1 (*cyp26b1*) as well as lecithin retinol acyltransferases (*lrata* and *lratb.1*) were strongly up-regulated in TTNPB treatments (cytochromes were down-regulated in DEAB treatments but not significantly). Also, dehydrogenase/reductase 3 (*dhrs3b*) was significantly up-regulated in TTNPB and down-regulated in DEAB treatments. Enzymes aldehyde dehydrogenase 1 family member A2 (*aldh1a2*) and *trans*-retinol 13,14-reductase (*retsat*) appeared only significantly down-regulated in TTNPB group. Enzymes all-retinol dehydrogenase 7 (*rdh7*) and $\beta ,\beta$-carotene 9’,10’-oxygenase-like (*bco2l*) were down-regulated in both treatments and the $\beta ,\beta$-carotene 15,15’-dioxygenase-like (*bco1*) and $\beta ,\beta$-carotene 9’,10’-oxygenase (*bco2a*) were up-regulated in DEAB treatments. In addition to these genes, Table 5 also presents a wide set of DETs related to thyroid axis, phototransduction signalling in retina, pigmentation-related genes, and matrix-related genes. Moreover, several transcription factors were also differentially expressed with DEAB and TTNPB treatments (Supplementary File 4).

## Discussion

The wide use of RNA-seq and the increasing number of studies focused on transcript-discovery or expression profiles are paving the way to obtain better transcriptomes and new assembling pipelines combining different bioinformatics tools, such as *CAFE*^22^, *TransFlow*^28^ or *RefShannon*^29^. These new pipelines have to deal with errors due to the high number of reads, weakly expressed or ‘uncommon’ transcripts, circular RNAs, gene fusions, and isoform discrimination. It was well established that assembly quality improves with longer paired-end reads^28^ but not increasing the input sequencing dataset^31^ due to, for example, the increase in low abundance transcripts and a plethora of unannotated transcripts, most of them containing intronic regions^32^. Hence, small sequencing datasets are desirable to decrease computational requirements and produce robust transcriptomes, since manual curation is excluded, even though it has been proved to render the best results^37^. Unfortunately, there is no consensus on the most efficient RNA-seq analysis protocols for characterizing and quantifying transcripts, which can result in a high number of TTs, such as the case of the Senegalese sole transcriptome^7^, with nearly 700,000 TTs and mapping rates higher than 90%^7,11^. These figures indicate an over-estimation of the real number of transcripts provoked by the huge amounts of reads in datasets used as input in the pipeline^31,32^ and the suboptimal assembling approach (Table 2). A partial solution to the challenging de novo assembling arrived from using only 27 high-quality sole libraries (Supplementary File 1) instead of all (111) replicates^7^, that represent about 23 % of the total number of reads, reduction that will not have any significant impact on the representativity of transcript sequences, even though a small overall decrease in biological variability might occur. Then, de novo assembling reduced RAM demands and time requirements, and reduced to 96 the former 180 assemblies produced by *TransFlow* workflow^28^ after considering only scaffolded sequences with a minimal coverage of 10 paired-end reads and increasing the *k*-mer sizes. The resulting transcriptomes had lower amounts of TTs with a lower proportion of “Unknown nature” (Table 2) in agreement with a recently published study where depth effects on de novo RNA-seq assembling was demonstrated^32^. The resulting high-quality, annotated transcriptome named SOLSEv5.0^34^ contained Illumina and GS-FLX libraries from different tissues (Table 1) and was consistent with other species where the presence of GS-FLX reads improved the final transcriptome^28^, as can be seen in Fig. 3 and Table 4. Therefore, the new assembling pipelines with less input datasets contributed to obtain more accurate “raw” transcriptomes than the original v4.0 approach (Table 2)^7,22,28^.

The numbers shown in Table 2 indicated that additional polishing was required besides published criteria^7^ of sequence similarity, gene orthology or coding prediction that improved the original v4.0^10,11^. In the present study, removal of low confidence TTs based on structural annotation and their mapping patterns (Table 3) diminished the huge amount of artefactual single-exon and uninformative TTs (Fig. 1), as well as TT redundancy (Fig. 2), which resulted in more robust mapping when using “definitive” Min2_Oases_Cap3 (Fig. 3). The new transcriptome SOLSEv5.0 (Table 4) contained 22,684 TTs coding for 17,429 different proteins, a reasonable number compared with phylogenetically near species such as the 21,516 different proteins described on *Cynoglossus semilaevis* genome^3^ or the 21,787 in Japanese flounder^1^. Based on SOLSEv5.0 features, it is proposed that any de novo transcriptome should rely on selecting a reasonable number of paired-end reads to be assembled with an optimised workflow, and a final removal of low confidence TTs (Fig. 3). In fact, a suboptimal assembly presents many low confidence TTs that are difficult to remove (Fig. 1), and a good assembling approach without polishing still retains many redundant TTs (Fig. 2).

But, even if de novo assembled transcriptomes are acceptable, bioinformatic tools for RNA-seq are not specifically designed for them due to the low annotation rates and biases in FDR corrections than are exacerbated by redundant transcripts^27^. This is why improved R scripts of *DEGenes Hunter* were developed in this study to analyse differential expression patterns using SOLSEv5.0^34^ as reference. The analysis relied on “prevalent” DETs (those TTs predicted as differentially expressed in all algorithms applied) that were then used for subsequent functional analyses (Supplementary File 4). The new features for functional interpretation in *DEGenes Hunter* provide dependable and accurate information about samples and functional changes (Figs. 4, 5, Supplementary File 4).

**Figure 7:**
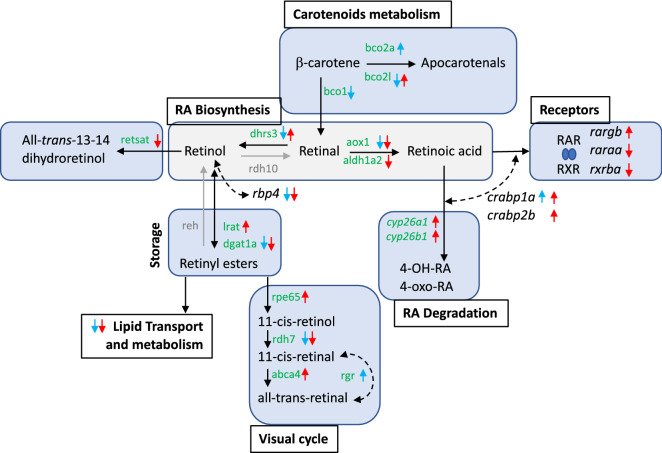
RA Metabolic pathways affected by DEAB and TTNPB treatments. Pathway names are indicated in bold and boxed, and the specific reactions and metabolites are enclosed in blue. Gene names are indicated in green. Arrows near the genes indicate up- or down-regulation, respectively, in DEAB (blue) or TTNPB (red) with respect to CTRL. Arrows and names in grey refer to non-modified pathways. Dashed lines indicate that the action could be in different pathways or metabolic steps. Drawing prepared by authors using Microsoft *PowerPoint*.

RA synthesis, degradation and cellular transport are highly conserved in vertebrates^38^. A coordinated action of these three pathways have revealed as essential to fine-tune RA levels gradients that in turn control morphogenesis and many other developmental processes. A previous study in sole using DEAB an TTNPB drugs demonstrated that RA levels modify the expression of some enzymes involved in retinol-retinal-RA conversion and RA degradation by establishing a negative feedback mechanism^39^. The present study demonstrates that acute exposure of larvae to these two drugs triggered an intense, specific and transient response at 24 h but with hardly any differences after 48 h. Although these treatments modified the expression of a common set of transcripts, the overall number of DETs were representative of drug-specific regulatory activities as demonstrated by PCA analysis (Fig. 4). Remarkably, both DEAB an TTNPB treatments activated a homeostatic response related to disturbance of retinoid metabolism (Figs. 5, 6). Key enzymes such as *dhrs3b* (Table 5), involved in the biosynthetic pathway and controlling the reduction of retinaldehyde back to retinol, appeared regulated through a negative feedback, promoting the synthesis of retinal in DEAB and retinol storage in TTNPB treatments. Moreover, the RA-receptor activation by TTNPB modified several pathways related to RA biosynthesis and degradation, retinol storage, carotenoid metabolism and visual cycle (Table 5, Fig. 7). Cytochromes *cyp26a1* and *cyp26b1* mRNAs were highly up-regulated at 24 and 48 h, concomitant with the induction of cellular RA binding proteins 1 and 2 (genes *crabp1* and *crabp2a* in Table 5, Fig. 7) to prevent excessive amounts of cellular all-*trans* retinoic acids (atRAs)^39–41^. Moreover, down-regulation of *aldh1a2* expression (Table 5, Fig. 7) indicated a switch-off of the second step of RA biosynthesis. Simultaneously with controlling the balance between RA production and degradation, other closely related pathways were modified to promote the retinol storage by increasing *lrat*, reducing adipogenesis by *retsat* (Table 5, Fig. 7)^42^, shifting $\beta$-carotene transformation toward apocarotenals instead of retinal production, and promoting 11-*cis* retinoids synthesis^43,44^. Unlike TTNPB, DEAB treatments modified specifically only those pathways related to retinal supply (carotenoids) and transformation (visual cycle). On the whole, TTNPB and DEAB treatments triggered a wide homeostatic response beyond biosynthesis and degradation, as a feedback mechanism to control atRA-mediated actions.

Concerning morphological aberrations, skeletogenesis disorders and malpigmentation, they were systemically associated with an excess of dietary vitamin A levels and external treatments with atRA. The present study indicates that increased RA signalling activated some pathways related to morphogenesis and collagen fibril organisation (Table 5). This is consistent with the fact that several transcription factors increased (*hoxb1, hoxb5, Hox-B6b, Hox-C5a, hoxc6, intestine-specific homeobox* and *six2*) or reduced (*hoxa13, lhx2, hnf1b, hmbox1, cux1, zfhx3* and *meis2*) their expression levels (Supplementary File 4). Going into detail, RA-mediated dysregulation of *hox* genes in zebrafish was teratogenic due to a modification of the anteroposterior developmental patterning^45,46^. High levels of RA that induced overexpression of *hox5b* provoked severe cardiac abnormalities^46,47^. Moreover, a zebrafish defective in *aldh1a2* that phenotypically lacks forelimbs (pectoral fins) and posterior branchial arches reduced the expression of *hoxb5a, hoxb6a* and *hoxb6b* along the entire length of its spinal cord expression domain, decreased *hoxb4a* expression in the hindbrain and failed to express *dlx2*, an early marker of apical ectodermal ridge activity in the fin bud^48^. In addition to transcription factors, TTNPB also modified the expression of 11 collagen-encoding genes (Table 5) and osteocalcin-2 (*bglap*). Although our experimental design was intended to evaluate the effects under a short-time drug exposure, this wide response of regulatory transcriptional factors and collagens does not preclude major structural alterations and agrees with the morphological aberrations found in larvae fed high levels of vitamin A^19,49^.

RA has also been associated with malpigmentation in flatfish during metamorphosis, such as in Japanese flounder where high doses of 9-*cis*-RA promoted development of adult chromatophores when supplied at the beginning of metamorphosis^50^. More recent studies demonstrated that an RA gradient is required for the generation of asymmetric pigmentation in flatfish with higher concentrations of atRA and 9-*cis*-RA in the ocular side than blind side^1^. In sole, previous studies failed to demonstrate an association between dietary levels of vitamin A and pseudoalbinism^49,51^, more related to dietary arachidonic acid levels^52^. In the current study, TTNPB results in up-regulation of GTP cyclohydrolase I (*gch1*; Table 5), the first and rate-limiting enzyme of pteridine synthesis and specifically expressed in xanthoblasts and to some extent in melanoblasts^53,54^. Moreover, TTNPB also modified enzymes modulating hypoxanthine biosynthesis (the main pigment in iridophores) such as *nt5c2* and *pnp5a*, suggesting disrupted pigmentation patterns in sole by modifying xanthophores and iridophores physiology^13,55^. High expression of *gch1* and disruption of iridophores, contrary to the pattern observed in pseudoalbino^13^, could be behind of the high rates of ambicolouration disorders currently found in the sole industry. Further experiments with longer evaluation periods will be necessary to demonstrate this hypothesis.

RA and TH establish a cross talk to regulate morphogenesis and cell development and differentiation in vertebrates mediated by the competition of the thyroid receptor (TR) and RA receptor (RAR) to form a heterodimer with the retinoid X receptor (RXR) for binding to gene promoters^1,56^. This interaction is crucial in flatfish metamorphosis governed mostly by THs^17,18,25^. Recent molecular findings in Japanese flounder have demonstrated that disorders in eye migration during metamorphosis are mediated by the RA-TH interaction: atRA reduced the proliferation of cells in the suborbital area of the blind side eye by up-regulating *rara* and down-regulating *thraa* and *thrb1*^1^. In sole, dietary vitamin A levels also modified their thyroid follicle number and size, T3 and T4 immunoreactive staining, skeletogenesis and mineralisation^19,49^. Moreover, *thrb* and *thrab* expression was reduced in metamorphic larvae fed with high levels of vitamin A^19^, and hormonal treatments demonstrated that atRA and TTNPB increased *thrb* mRNA levels and reduced *thrab* transcript levels in post-metamorphic larvae but not in pre-metamorphosis^39^. In the present study, metamorphic larvae exposed to TTNPB down-regulated the expression of *tg* and *thrab* (Table 5), but did not modify the expression of *dio2* and *thrb*, two genes that play a pivotal role in the asymmetric remodelling of sole head^18^. These data indicate a time-sensitivity window to hormonal treatments that might explain the minor effects on settlement and eye migration in sole, and the differences in gene expression patterns^19,49^.

DEAB and TTNPB modified the expression of a common set of 755 TTs (Fig. 4A), most of them at 24 h after drug treatments. These TTs were mainly related to “lipid metabolic process”, “lipid transport” and “lipid homeostasis”, processes that play a key role in the absorption and distribution of vitamin A. It is already known that high levels of retinal or RA inhibit adipogenesis when administered at early stages of adipocyte development^57^. External supply of retinal or *Raldh1*$^{-/-}$ mice mutants that accumulate retinal down-regulate the expression of adipogenic target genes and the transport protein rbp4 (also observed in the present study) through inhibiting the PPAR-$\gamma$:RXR dimer as a defensive mechanism to limit a RA excess in tissues^57^. Similarly, liganded RA-receptor or agonists inhibit adipogenesis by blocking C/EBP$\beta$-mediated induction of downstream genes^58^. Involvement of PPAR-$\gamma$ and C/EBP in adipogenesis regulation seems to be a conserved setting in fish^59,60^. The regulation of lipid transport, including several apolipoproteins involved in chylomicrons and very-low-density lipoprotein formation and lipases, is a key regulatory mechanism in the intestine and liver of pelagic and metamorphosing sole larvae to fit nutrient levels^10,61–64^. Therefore, our data suggest that DEAB and TTNPB treatments provided sensing signals to activate mechanisms that limit the availability of this fat-soluble vitamin, high levels of retinal in DEAB and enhanced RA-signalling in TTNPB treatments, which is consistent with the capacity of sole larvae to mobilize and store dietary vitamin A surplus^20^. They also support a negative feedback for vitamin A intestinal absorption and storage as previously suggested^44^ and highlight non-specific RA effects associated with lipid metabolism in DEAB and TTNPB treatments that should be taken into consideration when these drugs are used in fish trials.

In conclusion, even if new sequencing data using long read technologies (Oxford Nanopore or PacBio Sequel) would benefit any transcriptome assembling, re-analysis of already existing data^65^ is essential to provide optimised genomic tools for gene expression estudies and to exploit previous research investments. Hence this study demonstrates the usefulness of in silico strategy to generate the new SOLSEv5.0 as reference transcriptome for RNA-seq studies in sole. Also, new *DEGenes Hunter* capabilities have provided full relevant information for gene expression analyses. Both of them facilitated investigation into RA signalling and metabolism in larvae, revealing that DEAB and TTNPB drugs trigger a wide, coordinated and specific homeostatic response to maintain physiological RA levels. Moreover, expression changes of transcripts involved in morphogenesis and pigmentation also support a RA role in tissue remodeling that could be behind the morphological abnomalities associated with the supply of vitamin A in sole larvae. The identification of DETs shared by both drug treatments demonstrate non-specific drug effects related to lipid metabolism and gut absorption that it is relevant for the design of future studies using these drugs.

## Methods

### Computational infrastructure

Picasso supercomputer of University of Malaga was used for all bioinformatic tests, implementations and executions. It consists of an OpenSUSE LEAP 42.3 with Slurm queue system and Infiniband network (54/40 Gbps) containing 216 nodes with Intel E5-2670 2.6 GHz cores for a total of 3 456 cores and 22 TB of RAM. The required software was already installed, and all workflows and pipelines developed here were based on the workflow manager *AutoFlow* ^66^. Assembling workflow required the installation of *TransFlow* and its dependencies^28^, such as *Oases, SOAPdenovotrans, Minimus2, MIRA4, EULER-SR, CAP3, BUSCO, CD-HIT-EST*, and *FactoMineR*.

### Sequencing data and mapping

A total of 111 Illumina RNA-seq libraries of $2\times 75$ nt per read ($>1800$ million reads) and 5,663,225 GS-FLX RNA-seq single-end reads of Senegalese sole were available from BioProjects PRJNA255461, PRJNA241068 and PRJNA261151^7^. These datasets comprised nine experiments that included negative control and drug treatment in triplicate libraries, or even time-course responses. To optimize the number of reads to assemble, only one out of each biological replicate was randomly selected by experiment, and when a time series was available, only the first and the last sampling points were chosen (Supplementary File 1). Libraries containing GS-FLX reads were pre-processed using *SeqTrimNext* (based on *SeqTrim*^67^), while *SeqTrimBB* (based on *BBmap* suite^68^) was used for Illumina reads. Default parameters were applied in both cases. Illumina reads shorter than 60 bp were discarded, whereas the threshold for GS-FLX reads was set in 90.

Pre-processed reads (Supplementary File 1) were mapped onto the *S. senegalensis* genome draft^33^ using *Bowtie2*^69^ with the –no-mixed parameter to discard reads from unpaired alignments. *SAMtools*^70^ was used for mapping manipulation and read counts.

### De novo assembling and annotations

Pre-processed (useful) reads from libraries in Supplementary File 1 were assembled using the automated and modular framework *TransFlow*^28^ customised as follows: (i) increased *k*-mer length to 45 and 55 for Illumina reads (*TransFlow* variable $kmers = [45;55]), and to 29 for GS-FLX reads; (ii) removal of Illumina scaffolds with low coverage ($$<10\times$$, new *TransFlow* variable $NT_COVERAGE_IN_CONTIG=10); (iii) primary, intermediary assemblies are not considered, retaining only scaffolded assemblies; and (iv) additional *CD-HIT-EST*^71^ execution at the end of module 3 was included using options -c 0.95 -s 0.7 to reduce contig/scaffold redundancy at 95 % identity and 70 % overlap. The customised workflow is available at GitHub (https://github.com/JoseCorCab/TransFlow) and resulted in up to 96 assemblies. The *Danio rerio* GRCz11 build was used as reference transcriptome to select the best Senegalese sole “raw” transcriptome based on PCA distances. Sequencing reads ERR216329 from the SRA repository were used for quality assessment of the *D. rerio* transcriptome within *TransFlow*.

Structural status and functional assignment were annotated using *Full-LengtherNext* (P. Seoane, N. Fernández-Pozo and M.G. Claros, in preparation; http://www.scbi.uma.es/fulllengthernext for web execution, http://www.scbi.uma.es/site/scbi/downloads/313-full-lengthernext for instructions and off-line installation) configured to use UniProtKB full-length protein sequences for *Actinopterygii* taxon as in 24-09-2018 for first annotation database, and the *Vertebrata* division as in 24-09-2018 for the main database.

### Removal of low confidence transcripts

The rationale of the following processes is to sieve abnormal TTs. Firstly, a “raw” transcriptome was mapped against the preliminary *S. senegalensis* genome draft^33^ developed in our laboratory^8,9^ using *Minimap2*^72^ in splice mode, including option -uf for finding canonical splicing GT-AG sites, and option -C5 for a penalty of 5 for non-canonicals. The resulting SAM (Sequence Alignment Map) file was processed using SAMtools view -h -F 2052 to retain the best mapped position of multimapping transcripts without unmapped ones. According to Patterson et al.^32^, unspliced, fragmented, or missasembled, transcripts, as well as contaminant DNA, were then removed^32^, as well as TTs with ungapped mapping with $>90$ % identity.

The last polishing step was intended to retain those TTs having $>70$ % identity covering $>70$ % of its length to avoid fragmented or chimeric TTs containing too many intronic sequences. Since coverage and identity are not given in SAM files, SAM format was converted to PAF (Pairwise mApping Format; https://github.com/lh3/miniasm/blob/master/PAF.md) using sam2paf option of paftools.js (a script provided by *Minimap2* authors in https://github.com/lh3/minimap2/tree/master/misc). CIGARs (Concise Idiosyncratic Gapped Alignment Report) were also transfered using an in-house script written in Ruby (v2.4.1) merge_paf_cigar.rb (this and other postprocessing scripts are available at GitHub https://github.com/JoseCorCab/TransFlow_postprocessing). *Minimap2* artefacts produced when aligning transcripts to genomes were trimmed using paf_report.rb, which also calculates coverage, identity and exon number for each transcript.

### Fish trial and drug treatments

To investigate the effects of retinoic acid (RA) on sole larvae, fertilized eggs were obtained from naturally spawning Senegalese sole broodstock (IFAPA Centro El Toruño). The eggs were collected in the morning (8.00 am) and separated by buoyancy to get the fecundated fraction. Eggs were incubated in 15 L cylindro-conical tanks using a flow-through sea water circuit with gentle aeration at an initial density of 3000 embryos $\hbox {L}^{-1}$ and full exchange of sea water every hour. Newly hatched larvae (1 day post-hatch (dph)) were transferred to a 400 L tanks at an initial density of 45 to 50 larvae $\hbox {L}^{-1}$ and maintained in the dark until the onset of external feeding (3 dph). Detailed protocol for larval rearing was as previous described^73^. Shortly, larvae were supplied rotifers (*Brachionus plicatilis*) enriched for 3 h with *Tisochrysis lutea* (T-iso strain) from 3 till 9 dph. Moreover, *Artemia metanauplii* enriched for 24 h with *T. lutea* were supplied from 7 dph until the end of the experiment. Live microalgae *Nannochloropsis gaditana* and *T. lutea* were also added directly to water to enriched the live preys in the tank. During the trial, a 16 h:8 h light:dark photoperiod was used (light intensity 500 lux) and the mean water temperature and salinity was $21.1 \pm 0.3~^{\circ }$C and $34.2 \pm 0.2$ ppt, respectively. When larvae had just started the metamorphosis (12 dph), they were distributed into nine 15 L cylindro-conical tanks at the same initial larval density. Four days later (16 dph), when larvae were at the metamorphosis climax (metamorphic stages S2–S3)^17^, the following treatments were applied: (i) control group (CTRL) with dimethylsulfoxide (DMSO) (Sigma-Aldrich, Ref. 472301); (ii) TTNPB group with 4-[(*E*)-2-(5,6,7,8-tetrahydro-5,5,8,8-tetramethyl-2-naphthalenyl)-1-propenyl]benzoic acid (25 nM in DMSO, Sigma-Aldrich, Ref. T3757), a RA analog which acts as a selective agonist of RA-receptor; and (iii) DEAB group with 4-diethylaminobenzaldehyde (50 µM in DMSO, Sigma-Aldrich, Ref. 31830), an inhibitor of aldehyde dehydrogenase (ALDH) enzymes that prevents RA synthesis. Doses were selected according to previous data in sole^39,74^. Larvae were sampled at 24 and 48 h after the drug treatments, euthanized using MS-222, washed using diethylpyrocarbonated water, frozen in liquid nitrogen, and stored at $-\,80~^{\circ }$C until analysis.

The study has been carried out in accordance with EC Directive 86/ 609/EEC for animal experiments and Spanish regulations on animal welfare. All procedures were approved by the Animal Ethics Committee of IFAPA.

### RNA preparation

Pools of larvae (*n* = 5) from each tank (*n* = 3) were homogenised using the Fast-prep FG120 instrument (Bio101) and Lysing Matrix D (Q- Bio-Gene) for 40 s at speed setting 6. The numbers of embryos/larvae in the pools were always similar between conditions and replicates. Total RNA was isolated using the RNeasy Mini Kit (Qiagen) and treated twice with DNase I using an RNase-Free DNase kit (Qiagen) for 30 min to avoid genomic DNA contamination. Illumina libraries were constructed using mRNA-Seq sample preparation kit and sequenced using TruSeq SBS Kit v3-HS, in paired end mode, $2 \times 76$ bp in a fraction of a lane (1/6) of a HiSeq2000 sequencing system (Illumina, Inc) following the manufacturer’s protocol as previously reported^7^.

### Gene expresion analyses

Useful reads from Supplementary File 3 were mapped onto a reference transcriptome using *Bowtie2* with default parameters and –no-unal –no-mixed options to only retain appropriately mapped paired-end reads in the SAM file. The SAM file was sorted and converted to BAM by *SAMtools* sort and reads mapped per transcript were counted by *sam2counts* (https://github.com/vsbuffalo/sam2counts). Then, differential expression analysis was performed using an improved version of *DEGenes Hunter*^30^ available at https://github.com/seoanezonjic/DEgenesHunter. The first script degenes_Hunter.R is for differential expression using *edgeR, DEseq2, limma* and *NOISeq*, and generates files with quality control, expression data and a final report (DEG_report.html) where analysis details and plots are given. A DET is qualified as “prevalent” when it is differentially expressed in all algorithms used, and “possible” when not all algorithms qualify it as DET. By default, thresholds for a DET are absolute fold-change (FC) $>2$ and false discovery rate (FDR) $<0.05$ in every algorithm. The output includes a mean logFC and a combined FDR. More detail is given at its GitHub page.

Functional interpretation is launched with script functional_Hunter.R based mainly on *topGO* and *clusterProfiler*^75^ and then a final report (functional_report.html) is generated. Gene Ontology terms, and Reactome and KEGG databases for pathways are inspected to graphically display KEGG over-representation and clustering, GO over-representation analysis for the three hierarchies, and Reactome over-representation. SOLSEv5.0 DETs were interpreted using their zebrafish orthologues. Supplementary File 4 contains the most relevant results produced by *DEGenes Hunter* for the RA signalling study.

Additional biological interpretation of functional information based on Gene Ontology terms and KEGG/BioCarta pathways of *D. rerio* orthologues were carried out using the *ClueGO* plug-in^76^ for *Cytoscape*^76^ to visualise functionally grouped terms. PCA analyses with normalised, prevalent DETs in at least one of the four comparisons were performed using *ClustVis*^77^. Venn diagrams were performed with *InteractiveVenn* at http://www.interactivenn.net.

## Supplementary information

Supplementary files 1-8.

Supplementary file 4.

Supplementary  file 5.

## Data Availability

RNA-seq datasets are available at accession numbers SRR4897845, SRR1030352, SRR1282039, SRR2072478, DRR003148, SRR954861, SRR1282039, SRR100067, SRR2005826 as well as BioProjects 392999, PRJNA287107, 392587 and PRJNA392589. The genome draft of a female Senegalese sole is available at FigShare^33^; it includes five files: Sosen1_genome_scaffolds.fasta containing every contig and scaffold identifier and sequence in fasta format; Sosen1_genome_annotation.gff3 corresponding to a tentative annotation of genome contigs and scalffolds using MAKER2 and transcript sequences in SOLSEv5.0^34^; Sosen1_maker.transcripts.fasta containing the deduced transcripts from the gff3 annotation file; Sosen1_maker.proteins.fasta containing the deduced amino acid sequence for all deduced transcripts; and Sosen1_maker.proteins_annotation.tsv containing more annotations for deduced transcripts and proteins, such as transcript and protein lengths, best UniProtKB orthologue with identity % and E-value, structural status, open reading frame location in the transcript, description, GOs, KEGG codes, InterPro IDs, Pfam, EC and Unipathway, as tab-separated values (tsv format). *Full-LengtherNext* can be executed at http://www.scbi.uma.es/fulllengthernext; instructions and off-line installation can be obtained in http://www.scbi.uma.es/site/scbi/downloads/313-full-lengthernext. *TransFlow* can be downloaded from https://github.com/seoanezonjic/TransFlow; the customisation for this work can be downloaded from https://github.com/JoseCorCab/TransFlow. The new *DEGenes Hunter* version is available from https://github.com/seoanezonjic/DEgenesHunter. Other in-house scripts can be downloaded from https://github.com/JoseCorCab/TransFlow_postprocessing. SOLSEv5.0 transcriptome is available at FigShare^34^, including three files: SOLSEv5.0.fasta containing every TT identifier and its sequence in fasta format; SOLSEv5.0_ORTH_Drerio.tsv including the zebrafish orthologue (Subject_id) for every TT (Query_id) and its description as tab-separated values (tsv); and SOLSEv5.0_annot.tsv containing more annotations for each TT, such as its length, UniProtKB orthologue with identity % and E-value, structural status, open reading frame location in the TT, description, GOs, KEGG codes, InterPro IDs, Pfam, EC and Unipathway, in tsv format.

## References

[1] Shao C (2017). The genome and transcriptome of Japanese flounder provide insights into flatfish asymmetry. Nat. Genet..

[2] Alves RN (2016). The transcriptome of metamorphosing flatfish. BMC Genomics.

[3] Chen S (2014). Whole-genome sequence of a flatfish provides insights into ZW sex chromosome evolution and adaptation to a benthic lifestyle. Nat. Genet..

[4] Figueras A (2016). Whole genome sequencing of turbot (*Scophthalmus maximus*; Pleuronectiformes): a fish adapted to demersal life. DNA Res..

[5] Houston RD (2020). Harnessing genomics to fast-track genetic improvement in aquaculture. Nat. Rev. Genet..

[6] APROMAR. La Acuicultura en España 2019 v 1.3. Tech. Rep., Asociación Empresarial de Acuicultura de España (2019).

[7] Benzekri H (2014). De novo assembly, characterization and functional annotation of Senegalese sole (*Solea senegalensis*) and common sole (*Solea solea*) transcriptomes: integration in a database and design of a microarray. BMC Genomics.

[8] Manchado M, Planas JV, Cousin X, Rebordinos L, Claros MG (2016). Genomics in Aquaculture, chap. Current Status in Other Finfish Species: Description of Current Genomic Resources for the Gilthead Seabream (Sparus aurata) and soles (Solea senegalensis and Solea solea).

[9] Manchado M, Planas JV, Cousin X, Rebordinos L, Claros MG (2019). The Biology of Sole, Chap. Genetic and Genomic Characterization of Soles.

[10] Hachero-Cruzado I (2014). Characterization of the genomic responses in early senegalese sole larvae fed diets with different dietary triacylglycerol and total lipids levels. Comp. Biochem. Physiol. D Genomics Proteomics.

[11] Fatsini E, Bautista R, Manchado M, Duncan NJ (2016). Transcriptomic profiles of the upper olfactory rosette in cultured and wild Senegalese sole (*Solea senegalensis*) males. Comp. Biochem. Physiol. D Genomics Proteomics.

[12] Montero D (2015). Dietary vegetable oils: effects on the expression of immune-related genes in Senegalese sole (*Solea senegalensis*) intestine. Fish Shellfish Immunol..

[13] Pinto PIS (2019). Understanding pseudo-albinism in sole (*Solea senegalensis*): a transcriptomics and metagenomics approach. Sci. Rep..

[14] Campos C, Valente LMP, Conceição LEC, Engrola S, Fernandes JMO (2013). Temperature affects methylation of the myogenin putative promoter, its expression and muscle cellularity in senegalese sole larvae. Epigenetics.

[15] Firmino J (2017). Phylogeny, expression patterns and regulation of dna methyltransferases in early development of the flatfish, *Solea senegalensis*. BMC Dev. Biol..

[16] Carballo C (2020). Microalgal extracts induce larval programming and modify growth and the immune response to bioactive treatments and lcdv in senegalese sole post-larvae. Fish Shellfish Immunol..

[17] Manchado M, Infante C, Asensio E, Planas JV, Cañavate JP (2008). Thyroid hormones down-regulate thyrotropin beta subunit and thyroglobulin during metamorphosis in the flatfish Senegalese sole (*Solea senegalensis* Kaup). Gen. Comp. Endocrinol..

[18] Campinho MA (2018). A thyroid hormone regulated asymmetric responsive centre is correlated with eye migration during flatfish metamorphosis. Sci. Rep..

[19] Fernández I (2017). Vitamin a affects flatfish development in a thyroid hormone signaling and metamorphic stage dependent manner. Front. Physiol..

[20] Boglino A (2012). Commercial products for Artemia enrichment affect growth performance, digestive system maturation, ossification and incidence of skeletal deformities in Senegalese sole (*Solea senegalensis*) larvae. Aquaculture.

[21] Morillon A, Gautheret D (2019). Bridging the gap between reference and real transcriptomes. Genome Biol..

[22] You B-H, Yoon S-H, Nam J-W (2017). High-confidence coding and noncoding transcriptome maps. Genome Res..

[23] Smith-Unna R, Boursnell C, Patro R, Hibberd JM, Kelly S (2016). TransRate: reference-free quality assessment of de novo transcriptome assemblies. Genome Res..

[24] Ferraresso S (2013). Exploring the larval transcriptome of the common sole (*Solea solea* L.). BMC Genomics.

[25] Louro B, Marques JP, Manchado M, Power DM, Campinho MA (2020). Sole head transcriptomics reveals a coordinated developmental program during metamorphosis. Genomics.

[26] Kettleborough RNW (2013). A systematic genome-wide analysis of zebrafish protein-coding gene function. Nature.

[27] Hsieh P-H, Oyang Y-J, Chen C-Y (2019). Effect of de novo transcriptome assembly on transcript quantification. Sci. Rep..

[28] Seoane P (2018). TransFlow: a modular framework for assembling and assessing accurate de novo transcriptomes in non-model organisms. BMC Bioinform..

[29] Mao S, Pachter L, Tse D, Kannan S (2020). Refshannon: a genome-guided transcriptome assembler using sparse flow decomposition. PLoS ONE.

[30] González Gayte I, Bautista Moreno R, Seoane Zonjic P, Claros MG (2017). DEgenes hunter—a flexible R pipeline for automated RNA-seq studies in organisms without reference genome. Genomics Comput. Biol..

[31] Hayer KE, Pizarro A, Lahens NF, Hogenesch JB, Grant GR (2015). Benchmark analysis of algorithms for determining and quantifying full-length mRNA splice forms from RNA-seq data. Bioinformatics.

[32] Patterson J (2019). Impact of sequencing depth and technology on de novo RNA-Seq assembly. BMC Genomics.

[33] Claros MG, Seoane P, Manchado M (2020). Sequences and annotations of a provisional genome draft of a Senegalese sole female. Figshare.

[34] Claros MG, Córdoba-Caballero J, Seoane P, Manchado M (2020). Sequences and annotations of SOLSEv5.0 transcriptome. Figshare.

[35] Bradnam KR (2013). Assemblathon 2: evaluating de novo methods of genome assembly in three vertebrate species. Gigascience.

[36] Wajid B, Serpedin E (2016). Do it yourself guide to genome assembly. Brief Funct. Genomics.

[37] Kanitz A (2015). Comparative assessment of methods for the computational inference of transcript isoform abundance from RNA-seq data. Genome Biol..

[38] Rhinn M, Dollé P (2012). Retinoic acid signalling during development. Development.

[39] Boglino A, Ponce M, Cousin X, Gisbert E, Manchado M (2017). Transcriptional regulation of genes involved in retinoic acid metabolism in *Senegalese sole* larvae. Comp. Biochem. Physiol. B Biochem. Mol. Biol..

[40] Hernandez RE, Putzke AP, Myers JP, Margaretha L, Moens CB (2007). Cyp26 enzymes generate the retinoic acid response pattern necessary for hindbrain development. Development.

[41] Adams MK, Belyaeva OV, Wu L, Kedishvili NY (2014). The retinaldehyde reductase activity of dhrs3 is reciprocally activated by retinol dehydrogenase 10 to control retinoid homeostasis. J. Biol. Chem..

[42] Schupp M (2009). Retinol saturase promotes adipogenesis and is downregulated in obesity. Proc. Natl. Acad. Sci. U.S.A..

[43] Harrison EH (1821). Mechanisms involved in the intestinal absorption of dietary vitamin A and provitamin A carotenoids. Biochim. Biophys. Acta.

[44] Widjaja-Adhi MAK, Lobo GP, Golczak M, Von Lintig J (2015). A genetic dissection of intestinal fat-soluble vitamin and carotenoid absorption. Hum. Mol. Genet..

[45] Marlétaz F, Holland LZ, Laudet V, Schubert M (2006). Retinoic acid signaling and the evolution of chordates. Int. J. Biol. Sci..

[46] Waxman JS, Yelon D (2009). Increased hox activity mimics the teratogenic effects of excess retinoic acid signaling. Dev. Dyn..

[47] Waxman JS, Keegan BR, Roberts RW, Poss KD, Yelon D (2008). Hoxb5b acts downstream of retinoic acid signaling in the forelimb field to restrict heart field potential in zebrafish. Dev. Cell.

[48] Grandel H (2002). Retinoic acid signalling in the zebrafish embryo is necessary during pre-segmentation stages to pattern the anterior-posterior axis of the CNS and to induce a pectoral fin bud. Development.

[49] Fernández I (2009). Effect of dietary vitamin A on Senegalese sole (*Solea senegalensis*) skeletogenesis and larval quality. Aquaculture.

[50] Miwa S, Yamano K (1999). Retinoic acid stimulates development of adult-type chromatophores in the flounder. J. Exp. Zool..

[51] Fernández I, Gisbert E (2010). Senegalese sole bone tissue originated from chondral ossification is more sensitive than dermal bone to high vitamin A content in enriched Artemia. J. Appl. Ichthyol..

[52] Villalta M, Estévez A, Bransden MP (2005). Arachidonic acid enriched live prey induces albinism in Senegal sole (*Solea senegalensis*) larvae. Aquaculture.

[53] Braasch I, Schartl M, Volff J-N (2007). Evolution of pigment synthesis pathways by gene and genome duplication in fish. BMC Evol. Biol..

[54] Nord H, Dennhag N, Muck J, von Hofsten J (2016). Pax7 is required for establishment of the xanthophore lineage in zebrafish embryos. Mol. Biol. Cell.

[55] Zhou L (2019). Genetic characteristic and RNA-Seq analysis in transparent mutant of carp-goldfish nucleocytoplasmic hybrid. Genes (Basel).

[56] Li H (2015). Ectopic cross-talk between thyroid and retinoic acid signaling: a possible etiology for spinal neural tube defects. Gene.

[57] Ziouzenkova O (2007). Retinaldehyde represses adipogenesis and diet-induced obesity. Nat. Med..

[58] Schwarz EJ, Reginato MJ, Shao D, Krakow SL, Lazar MA (1997). Retinoic acid blocks adipogenesis by inhibiting c/ebpbeta-mediated transcription. Mol. Cell Biol..

[59] Wafer R, Tandon P, Minchin JEN (2017). The role of peroxisome proliferator-activated receptor gamma (pparg) in adipogenesis: applying knowledge from the fish aquaculture industry to biomedical research. Front. Endocrinol. (Lausanne).

[60] Salmerón C (2018). Adipogenesis in fish. J. Exp. Biol..

[61] Roman-Padilla J, Rodríguez-Rua A, Claros MG, Hachero-Cruzado I, Manchado M (2016). Genomic characterization and expression analysis of four apolipoprotein A-IV paralogs in Senegalese sole (*Solea senegalensis* Kaup). Comp. Biochem. Physiol. B Biochem. Mol. Biol..

[62] Román-Padilla J, Rodríguez-Rúa A, Manchado M, Hachero-Cruzado I (2016). Molecular characterization and developmental expression patterns of apolipoprotein A-I in Senegalese sole (*Solea senegalensis* Kaup). Gene Expr. Patterns.

[63] Román-Padilla J, Rodríguez-Rúa A, Ponce M, Manchado M, Hachero-Cruzado I (2017). Effects of dietary lipid profile on larval performance and lipid management in Senegalese sole. Aquaculture.

[64] Roman-Padilla J, Rodríguez-Rúa A, Carballo C, Manchado M, Hachero-Cruzado I (2018). Phylogeny and expression patterns of two apolipoprotein E genes in the flatfish Senegalese sole. Gene.

[65] Kovalevskaya NV (2016). Dnadigest and repositive: connecting the world of genomic data. PLoS Biol..

[66] Seoane P (2016). AutoFlow, a versatile workflow engine illustrated by assembling an optimised de novo transcriptome for a non-model species, such as faba bean (*Vicia faba*). Curr. Bioinform..

[67] Falgueras J (2010). SeqTrim: a high-throughput pipeline for pre-processing any type of sequence read. BMC Bioinform..

[68] Bushnell, B. *BBmap Suite* (2014).

[69] Langmead B, Salzberg SL (2012). Fast gapped-read alignment with Bowtie 2. Nat. Methods.

[70] Li H (2009). The sequence alignment/Map format and SAM tools. Bioinformatics.

[71] Li W, Godzik A (2006). CD-HIT: a fast program for clustering and comparing large sets of protein or nucleotide sequences. Bioinformatics.

[72] Li, H. Minimap2: fast pairwise alignment for long DNA sequences. *arXiv* (2017). http://arXiv.org/1708.01492.

[73] Fernández-Díaz C (2001). Growth and physiological changes during metamorphosis of Senegal sole reared in the laboratory. J. Fish Biol..

[74] Ponce M (2011). Genomic characterization, phylogeny and gene regulation of g-type lysozyme in sole (*Solea senegalensis*). Fish Shellfish Immunol..

[75] Yu G, Wang L-G, Han Y, He Q-Y (2012). clusterProfiler: an R package for comparing biological themes among gene clusters. OMICS.

[76] Bindea G (2009). ClueGO: a Cytoscape plug-in to decipher functionally grouped gene ontology and pathway annotation networks. Bioinformatics.

[77] Metsalu T, Vilo J (2015). ClustVis: a web tool for visualizing clustering of multivariate data using principal component analysis and heatmap. Nucleic Acids Res..

