# Supplementary material for: An improved de novo assembling and polishing of Solea senegalensis transcriptome shed light on retinoic acid signalling in larvae
Source: Sci Rep. 2020 Nov 26;10:20654. doi: 10.1038/s41598-020-77201-z (PMC7691524; doi:10.1038/s41598-020-77201-z)

Supplementary Files

Supplementary File 1 : Libraries selected for the new *de novo* assembling approach.

Information about the experimental treatment, library names, total amount of raw and useful reads, and their mapping rate onto a sole genome draft is included.

Experimental class	Experimental setting	Library code	Original reads	Useful reads (no.)	Discarded reads (no.)	Discarded reads (%)	Mapping rate (%)
Mixed tissues	Mixed tissues	GS-FLX	5 663 225	3 104 734	2 050 799	36.21	10.59
Metamorphosis	Larvae stage S0	H537	33 922 196	25 716 110	8 206 086	24.19	60.64
	Larvae stage S4	H550	31 807 020	25 543 201	6 263 819	19.69	59.34
Embryogenesis	Embryos gastrula	H552	31 042 548	24 614 521	6 428 027	20.71	63.96
	Embryo neurula-somitogenesis	H563	27 352 934	22 596 812	4 756 122	17.39	65.58
RA and TH regulation in embryos	Embryo Gastrula DMSO 3h	H565	22 401 658	18 634 806	3 766 852	16.82	62.43
	Embryos Gastrula ATRA 3h	H569	26 408 348	21 578 222	4 830 126	18.29	63.29
	Embryos Gastrula TTNPB 3h	H570	22 795 621	19 289 221	3 506 400	15.38	63.21
	Embryos Gastrula DEAB 3h	H574	29 401 472	24 871 553	4 529 919	15.41	62.87
	Embryos Gastrula TU 3h	H578	11 497 947	9 305 955	2 191 992	19.06	63.40
RA and TH regulation in metamorphic larvae (16 dph)	Larvae S2-S3 DMSO 24h	H579	9 090 312	7 147 461	1 942 851	21.37	65.39
	Larvae S2-S3 ATRA 24h	H583	8 706 819	6 707 482	1 999 337	22.97	68.09
	Larvae S2-S3 TTNPB 24h	H587	6 427 675	5 054 039	1 373 636	21.37	69.55
	Larvae S2-S3 DEAB 24h	H588	10 509 746	7 768 277	2 741 469	26.09	69.40
	Larvae S2-S3 TU 24h	H592	10 333 216	7 802 845	2 530 371	24.49	66.82
	Larvae S2-S3 DMSO 48h	H596	9 151 482	7 144 061	2 007 421	21.94	66.35
	Larvae S2-S3 ATRA 48h	H597	19 378 709	14 799 486	4 579 223	23.63	65.35
	Larvae S2-S3 TTNPB 48h	H601	9 905 767	7 759 228	2 146 539	21.67	66.96
	Larvae S2-S3 DEAB 48h	H605	11 539 156	8 602 016	2 937 140	25.45	68.66
	Larvae S2-S3 TU 48h	H606	9 856 909	6 708 121	3 148 788	31.94	64.28
TH and cortisol regulation in postlarvae (20 dph)	Postlarvae Methimazole 13d	H614	8 697 570	6 817 323	1 880 247	21.62	58.50
	Postlarvae mifepristone 13d	H615	7 092 632	5 492 226	1 600 406	22.56	57.09
RA regulation in postlarvae (21dph)	Postlarvae DMSO 14d	H619	10 196 687	8 577 874	1 618 813	15.88	59.70
	Postlarvae Iopanoic 14d	H623	8 753 415	7 227 151	1 526 264	17.44	55.56
Thermal regulation (19 dph)	Postlarvae grown at 20ºC	H625	7 126 353	5 824 671	1 301 682	18.27	62.79
	Postlarvae grown at 16ºC	H627	6 417 977	4 704 322	1 713 655	26.70	66.31
Larvae incubated at two salinities	Larvae 1 dph S10	H632*	24 451 407	19 850 709	4 600 698	18.82	67.02
	Larvae 1 dph S36	H634*	10 883 054	8 224 988	2 658 066	24.42	70.81
	Larvae 3 dph S10	H638*	9 684 921	7 591 469	2 093 452	21.62	72.51
	Larvae 3 dph S36	H639*	8 994 334	6 289 113	2 705 221	30.08	75.43

dph: days post-hatch. *: Libraries discarded for assembling, all of them from the same experimental batch PRJNA255461.

Supplementary File 2 : Length distribution of low confidence TTs removed from Table 3

Unmapping and atypical TTs were very short transcripts, with a median length ranging from 312 to 547 nt. Since interquartile range in unmapping and atypical TTs is wider for Oases_k45 than for Min2_Oases_Cap3, it was strongly suggested that some Oases_k45 fragments were completed TTs in Min2_Oases_Cap3 due to the presence of GS-FLX reads. In addition, this supported the inclusion of the Min2_Oases_Cap3 assembly in the comparison. However, low quality TT median lengths (2 359 bp for Oases_k45 and 1 894 bp for Min2_Oases_Cap3) were larger than for their corresponding "definitive" transcriptomes (2 010 bp for Oases_k45 and 1 236 bp for Min2_Oases_Cap3), indicating that low quality TTs may be consequence of misasembling, chimeric assembling, or excessive contig extension (remember their low identity with genome sequences in Table 3), supporting that they merit removal.

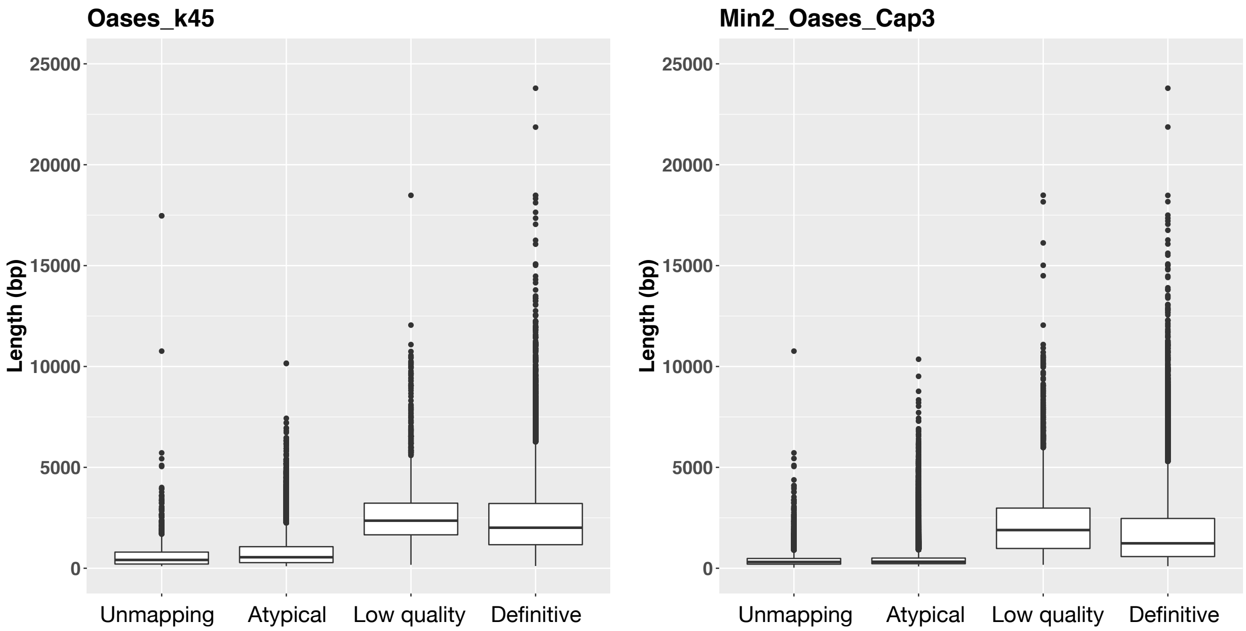

Supplementary File 3 : Summary of 18 Illumina libraries used in the study of RA signalling in metamorphosing larvae. These samples were available in the BioProjects mentioned in Materials and Methods and some of them also in Supplementary File 1.

Treatment duration	Experimental condition	Replicate	Sample ID	Raw reads	Useful reads
24 h	DMSO	1	H579	9 090 312	7 147 446
		2	H580	8 601 631	6 721 394
		3	H581	8 273 686	6 487 485
	DEAB	1	H588	10 509 746	7 768 262
		2	H590	8 957 246	7 148 521
		3	H589	6 561 553	4 824 372
	TTNPB	1	H585	6 255 481	4 838 137
		2	H586	8 064 663	6 362 387
		3	H587	6 427 675	5 054 049
48 h	DMSO	1	H594	10 921 347	8 556 995
		2	H595	5 805 820	4 495 229
		3	H596	9 151 482	7 144 049
	DEAB	1	H604	16 436 438	12 638 412
		2	H603	10 114 056	7 847 654
		3	H605	11 539 156	8 602 074
	TTNPB	1	H600	9 728 264	2 166 642
		2	H601	9 905 767	2 146 515
		3	H602	9 556 818	2 149 450

Supplementary File 4 : Complete reports when untreated samples (CTRL) are compared with treatments using TTNPB or DEAB for 24 and 48 h as provided by *DEGenes Hunter* using SOLSEv5.0 as transcriptome reference.

The file `DEGenesHunter_reports_SOLSEv5.0.zip` contains the following two files and five folders:

- File `Normalized_counts_DESeq2.tsv` includes the normalised counts as provided by *DESeq2* for the 33 467 TT that were expressed and for every analysed sample, as tab-separated values (tsv).
- File `prevalent_DESeq2_normalized_counts.tsv` contains the normalised counts as provided by *DESeq2* only for the 4 740 prevalent DETs in every analysed sample, in tsv format. It was necessary for the PCA plot in Figure 4B.
- Folder `CTRL_vs_DEAB_24h` contains files after untreated (CTRL) samples were compared to DEAB samples at 24 h. It includes the differential expression report (`DEG_report.html`) and the functional analysis report (`functional_report.html`) of the prevalent DETs. The file `CTRL_vs_DEAB_24h.tsv` is in tsv format, containing logFCs, FDRs and P-values for the four algorithms used; it also includes a column to indicate which algorithm tag the TT as DET ('TRUE'), combined FDR and P-values, and whether the gene is prevalent or not. File `CTRL_vs_DEAB_24h_annotated.tsv` includes the TT ID, the mean logFC from previous file, the ENSEMBLID for zebrafish orthologue, the gene name and a description, in tsv format.
- Folder `CTRL_vs_DEAB_48h` contains files after CTRL samples were compared to DEAB samples at 48 h. File meaning is as above.
- Folder `CTRL_vs_TTNPB_24h` contains files after CTRL samples were compared to TTNPB samples at 24 h. File meaning is as above.
- Folder `CTRL_vs_DEAB_48h` contains files after CTRL samples were compared to TTNPB samples at 48 h. File meaning is as above.
- Folder `FA_Common-Specific` contains tree functional reports regarding prevalent DETs of Figure 4A. `DEAB_24_specific.html` is for DETs that only appear at DEAB 24h. `TTNPB_24_specific.html` is for DETs that only appear at TTNPB 24h. Common DETs after treatment of DEAB at 24h and TTNPB at 24h are in `DEAB_common_TTNPB_24h.html`

Supplementary File 5 : Essential reports when untreated samples (CTRL) are compared with treatments using TTNPB or DEAB for 24 and 48 h as provided by *DEGenes Hunter* using "raw" v4.0 as transcriptome reference.

The file `DEGenesHunter_reports_v4.0.zip` contains the following file and folders:

- File `v4.0_counts.tsv` includes the counts for 336 816 TTs (48.3 %) that were mapped and for every analysed sample, as tab-separated values (tsv).
- Folder `CTRL_vs_DEAB_24h` contains files after untreated (CTRL) samples were compared to DEAB samples at 24 h. It includes the differential expression report (`DEG_report.html`) and the functional analysis report (`functional_report.html`) of the prevalent DETs . The file `Normalized_counts_DESeq2.tsv` is in tsv format, containing the normalised counts as provided by *DESeq2* only for the 71 610 expressed TTs (10.1 % of TTs) in this comparison. File `hunter_results_table_annotated.tsv` contains the results of the *DEgenes Hunter* analysis in tsv format, including the ENSEMBL ID of zebrafish, its Entrez code and the gene symbol, when available.
- Folder `CTRL_vs_DEAB_48h` contains files after CTRL samples were compared to DEAB samples at 48 h. File meaning is as above for the 73 217 expressed TTs (10.4 % of TTs).
- Folder `CTRL_vs_TTNPB_24h` contains files after CTRL samples were compared to TTNPB samples at 24 h. File meaning is as above for the 70 148 expressed TTs (10.0 % of TTs).
- Folder `CTRL_vs_DEAB_48h` contains files after CTRL samples were compared to TTNPB samples at 48 h. File meaning is as above for the 72 186 expressed TTs (10.3 % of TTs).

Supplementary File 6 **Overview of the differential expression analysis provided in Supplementary File 5 using "raw" v4.0 transcriptome as reference**

A: Distribution of DET counts after mapping using as reference SOLSEv5.0 (green) and "raw" v4.0 (orange). Data are shown by treatment (CTRL, DEAB and TTNPB) and sampling time (24 and 48 h). Asterisks denote significant differences using Mann Whitney test. B: Venn diagrams representing the number of DETs by DEAB and TTNPB treatments and by sampling time (24 and 48 h) with respect to CTRL (Control). Total amounts of DETs are indicated in parentheses. C: Principal component analysis (PCA) plot using DETs. As in Figure 4, it shows only three distinct clusters (enclosed by ellipses) that indicate CTRL (red), DEAB (blue) and TTNPB (green) treatments. Sampling points are identified with circles (24 h) and squares (48 h).

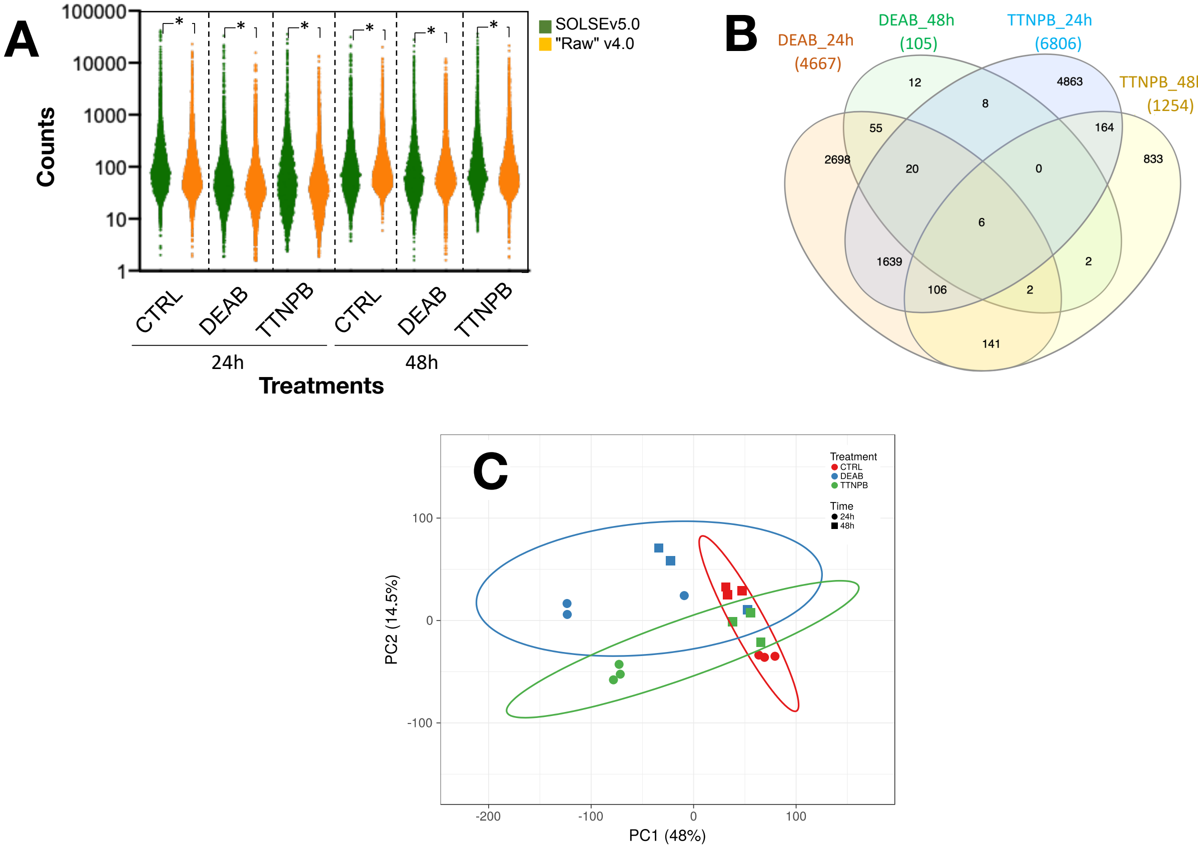

Supplementary File 7 Comparison of DETs obtained with SOLSEv5.0 and “raw” v4.0 transcriptomes as reference based on the ENSEMBL ID of their zebrafish orthologue

In the upper part, the Venn diagrams obtained with *InteractiveVenn* compare the number of DETs with a zebrafish orthologue for DEAB and TTNPB treatments at both sampling times compared to the CTRL. Total numbers of DETs are indicated in parenthesis. A comparison of the total number of DETs (global) is also shown, resuming that an important number of DETs (1 793) were revealed with both analyses, but a non-negligible amount of them were obtained depending on the reference transcriptome (1 174 exclusive of SOLSEv5.0 and 2 071 exclusive of “raw” v4.0).
In the lower part, the vulcano plots prepared with *Excel* showing only DETs reveal that more TTs are obtained for “raw” v4.0 but higher values of logFC and lower *P*-values usually correspond to SOLSEv5.0 DETs, suggesting that this transcriptome might confer more statistical power to the differential expression analysis.

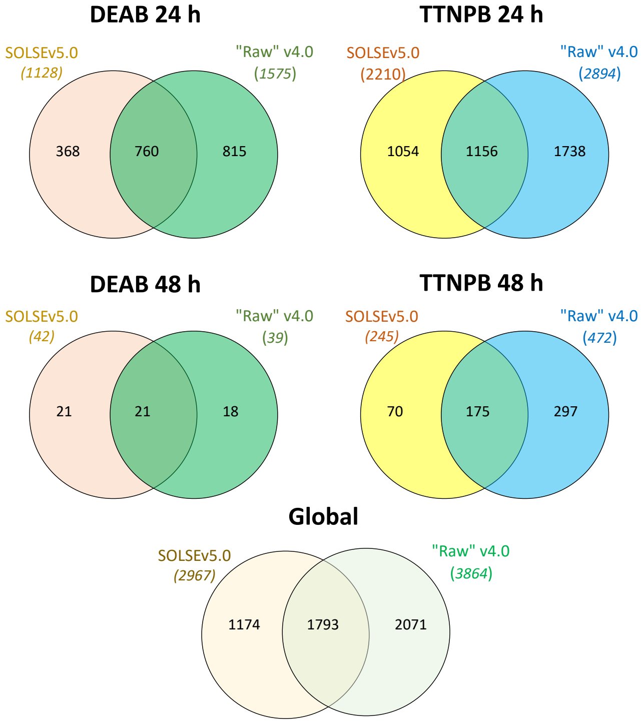

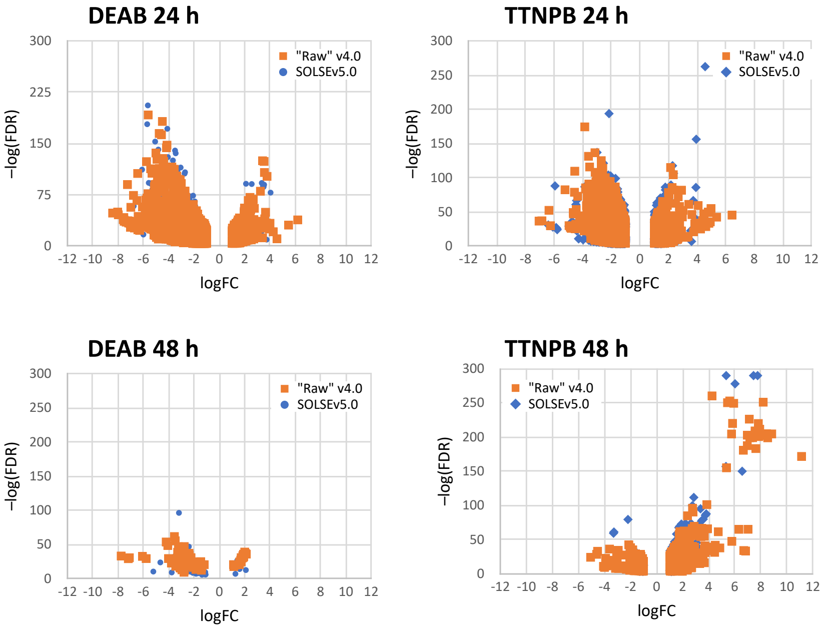

Supplementary File 8 Top 20 significant GOs obtained from the enrichment analysis of DETs identified in the DEAB and TTNPB treatments after 24 and 48 h from Supplementary File 5 using "raw" v4.0 transcriptome as reference

Categories were sorted by gene ratio (proportion of DETs present in the total amount of genes assigned to a GO category), represented by the X axis. Point sizes reflect the number of DETs on each category. Colours represent the adjusted *P*-value of each category. Categories that match with SOLSENv5.0 in Figure 5 are in red. '*': categories represented both in DEAB and TTNPB treatments. '†': categories represented at both times within the same treatment

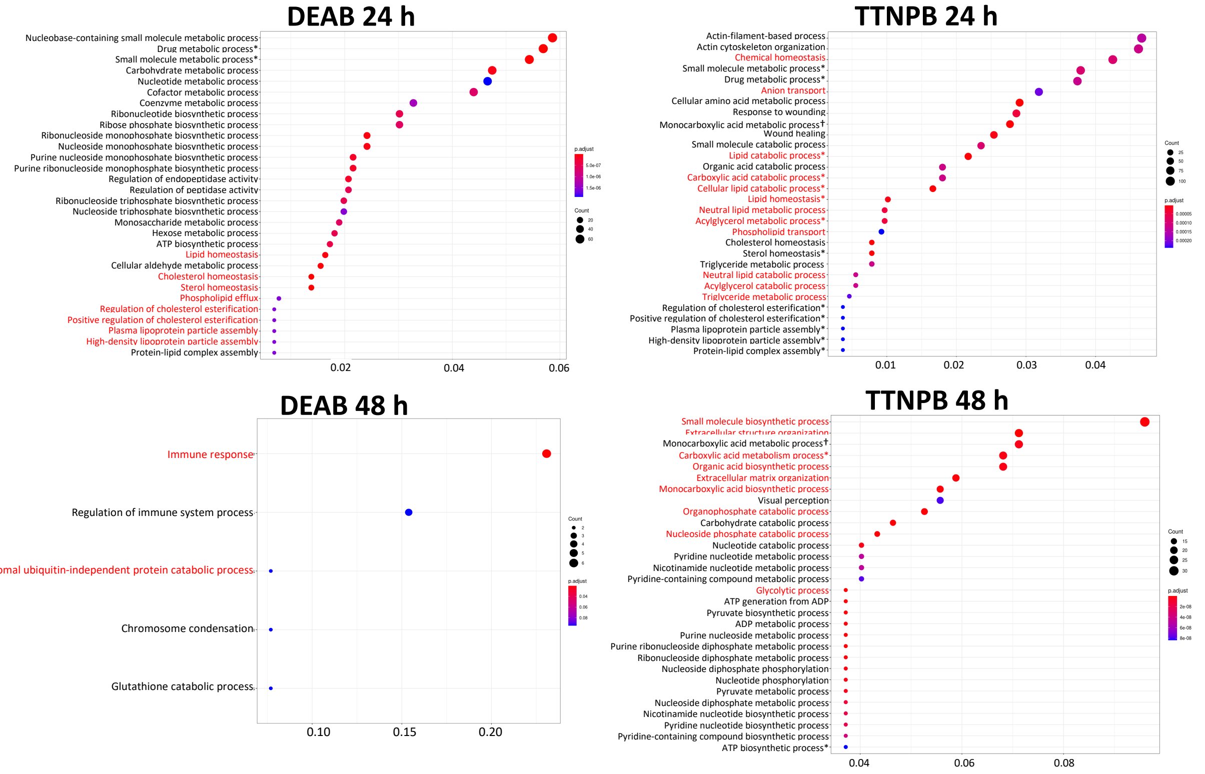

Supplement: Supplementary file 1 — Supplementary files 1-8. [file 41598_2020_77201_MOESM1_ESM.pdf]
