# Supplementary material for: An improved de novo assembling and polishing of Solea senegalensis transcriptome shed light on retinoic acid signalling in larvae
Source: Sci Rep. 2020 Nov 26;10:20654. doi: 10.1038/s41598-020-77201-z (PMC7691524; doi:10.1038/s41598-020-77201-z)
Supplement: Supplementary file 2 — Supplementary file 4. [file 41598_2020_77201_MOESM2_ESM.zip › CTRL_vs_TTNPB_24h/functional_report.html]

 

 

 

 
 
 


 


 /mnt/home/users/bio_267_uma/josecordoba/proyectos/lenguado/RA_DE/RA_all_comparisons/v5_transcriptome/DEA_ctrl_vs_TTNPB_24h/results/functional_report.utf8.md 

 
 
 
 
 
 
 
 
 
 
 
 
 
 

 
 
 


 


 


 

 

 

 


 

 


 

 


 


 
 
 
 
 
 

 


 


 


 
 
  DEgenes Hunter - Functional analysis report  
 
  Used data in this analysis  
 Specifically, in this experiment set, known experiment labels are: 
 
 [Control] ctrl_24h_1 
 [Control] ctrl_24h_2 
 [Control] ctrl_24h_3 
 [Treatment] trt_TTNPB_24h_1 
 [Treatment] trt_TTNPB_24h_2 
 [Treatment] trt_TTNPB_24h_3 
 
 
 
  General description   
 This report contains all the functional information that was requested by the options when functional_Hunter.R was executed. The functional categories can be: 
 
 KEGG pathways 
 GO:
 
 Biological Process 
 Molecular Function 
 Cellular Component 
  
 Reactome pathways 
 
 All the functional categories are computed with CluterProfiler and GO caterogires are computed also with TopGo. Some sections will not show if there are not sinficative results. Each category is analysed using Over representation analysis (ORA) and Gene Set Analysis (GSEA). The ORA method takes a group of significative DEGs (only DEGs, upregulated DEGs or downregulated DEGs) and performs a hypergeometric test for each term of the selected functional category. In the case of the GSEA method, all the genes are sorted by their fold-change and the algorithm scan which genes with similar fold-change shares a term of the selected functional category. 
 Statistics about input results obtained from DEGenes Expression Hunter are: 
 
 
 
 Var1 
 Freq 
 
 
 
 
 FILTERED_OUT 
 20885 
 
 
 NOT_DEG 
 26030 
 
 
 POSSIBLE_DEG 
 1230 
 
 
 PREVALENT_DEG 
 3203 
 
 
 
 
 
  KEGG analysis  
 
  Over Representation Analysis  
 The ORA method takes a group of significative DEGs (only DEGs, upregulated DEGs or downregulated DEGs) and performs a hypergeometric test for each term of the selected functional category. 
  Barplot  
 The plot shows the functional top significative terms in ascendant order by adjusted pvalue. The color represents the associated adjusted pvalue. The X axis represents the proportion of the known genes for a given functional term that are identified in the expression data. 
 
   
 
  Dotplot  
 The plot shows the top functional terms in descendant order by gene ratio. This ratio is the proportion of the known genes for a given functional term that are identified in the expression data. The color represents the associated adjusted pvalue. The X axis represents the gene ratio and the dot size the number of DEG genes associated to the functional term. 
 
   
 
  Gene-Concept Network  
 The network connects the top functional terms (brown nodes) to their associated genes (grey or colored nodes). The size of the functional terms shows the number of connected genes. 
 
   
 
  Enrich Map plot  
 The network connects the top functional terms (nodes) between them trought their associates genes (grey edges, thickness represents the number of shared genes.). The size of the functional terms shows the number of connected genes and the color the adjusted pvalue of the functional term. 
 
   
 
  Heatplot  
 Top functional terms are show in the Y axis whereas in the X axis are show the associated genes to them. The coloured squares indicates the fold change value. 
 
   
 
  Upsetplot  
 Top functional terms are listed the Y axis with the number of genes associated to them (Set size). Each column of dots marks one cluster of functional terms. The X axis indicates how many genes are shared exclusively for the members of one cluster. 
 
   
 
 
 
 
  ClusterProfiler KEGG table  
 Detailed table with the data obtained from KEGG pathways (ORA method only). For each pathway, the identifier, link to KEGG DB with the detected genes, name, adjusted pvalue and genes (count and ids) 
 
 
 
  
 ID 
 Description 
 p.adjust 
 Count 
 geneID 
 
 
 
 
 dre04910 
  dre04910  
 Insulin signaling pathway 
 0.0000144 
 46 
 mtor/g6pca.2/ppp1caa/pik3r1/fbp1b/grb2b/araf/akt3a/eif4ebp1/sorbs1/phkb/prkag2a/tsc2/prkab1b/prkar1ab/akt2/mapk3/flot2a/phka2/mapk1/prkcz/insra/gys2/gsk3b/foxo1a/si:ch211-79e4.4/prkaa2/trip10a/elk1/mknk2b/srebf1/irs2b/rps6kb1b/pik3r2/pck2/gck/pik3ca/prkacab/rptor/crk/hk1/prkci/hrasb/lipeb/crkl/hkdc1 
 
 
 dre04012 
  dre04012  
 ErbB signaling pathway 
 0.0000229 
 33 
 mtor/camk2b/plcg1/camk2a/camk2d2/pik3r1/abl2/camk2g1/grb2b/camk2g2/araf/akt3a/eif4ebp1/cdkn1a/pak2a/gab1/akt2/camk2d1/mapk3/mapk1/nck2b/gsk3b/si:ch73-383l1.1/elk1/rps6kb1b/camk2b1/pik3r2/pik3ca/erbb3a/crk/stat5a/hrasb/crkl 
 
 
 dre04144 
  dre04144  
 Endocytosis 
 0.0001478 
 67 
 tfr1a/rnf41l/rab5aa/igf2r/ap2m1a/cyth1a/arfgef1/sh3gl2a/hspa8/agap2/kif5ab/sh3gl1b/rhoab/arpc5b/cltcb/LOC563561/tgfbr1b/agap1/epn2/chmp4bb/cav1/arf2b/clint1a/vps4a/rab8a/vps37b/kif5ba/agap3/iqsec2b/arfgef2/eps15l1a/prkcz/zfyve16/asap1b/igf1ra/ist1/arf3b/rab7a/rab11fip2/ap2a1/stam2/arrb2a/iqsec1b/wasla/washc4/wipf2b/smad2/dnm2a/zgc:165502/hgs/pld2/acap1/zgc:194578/acap3b/rab4a/iqsec3b/pard3ab/capzb/chmp1a/prkci/hrasb/ap2b1/dnm1a/grk4/rab11al/grk3/pip5k1ca 
 
 
 dre04530 
  dre04530  
 Tight junction 
 0.0002546 
 50 
 myh9a/wu:fd14a01/actr2a/ezrb/itgb1a/actb1/actn1/rhoab/dlg1l/rapgef6/tuba8l2/tuba1b/tuba1c/dlg2/magi1b/cldn2/prkag2a/myhc4/cacna1da/prkab1b/dlg1/rab8a/myhb/si:ch73-61d6.3/si:ch211-191a24.4/prkcz/ppp2cb/cldn19/afdna/amotl2a/jam2a/prkaa2/rock1/cldn15lb/llgl2/cldn15la/cldn15a/ppp2r1ba/pard3ab/patj/slc9a3r1a/myh9b/scrib/prkacab/prkci/actn4/map3k5/oclnb/rapgef2/jam3b 
 
 
 dre04114 
  dre04114  
 Oocyte meiosis 
 0.0055605 
 34 
 camk2b/ppp2r5ca/camk2a/camk2d2/ppp1caa/slka/camk2g1/ppp3ccb/ppp2r5a/camk2g2/ywhaba/ywhae1/ppp2r5cb/camk2d1/mapk3/mapk1/anapc4/igf1ra/ppp2cb/ppp2r5d/ppp3cb/itpr3/ppp3cca/rps6ka3a/ppp2r1ba/ppp3r1b/camk2b1/cpeb2/cpeb4b/prkacab/cpeb4a/mapk12a/adcy9/ppp2r5b 
 
 
 dre04261 
  dre04261  
 Adrenergic signaling in cardiomyocytes 
 0.0055605 
 46 
 atp1a3b/slc8a1b/camk2b/cacna1fb/tpm1/ppp2r5ca/camk2a/camk2d2/ppp1caa/atp1a1a.4/camk2g1/ppp2r5a/camk2g2/akt3a/atp1b1b/atp1b1a/atp1a3a/ppp2r5cb/atp1b2b/cacna1da/gnaq/atp2a3/akt2/camk2d1/mapk3/si:rp71-17i16.5/mapk1/ppp2cb/cacng1b/ppp2r5d/cacna1sa/creb3l3a/si:dkey-28b4.8/ppp2r1ba/ppp2r3a/camk2b1/cacnb1/agt/prkacab/atp2a2b/myh7l/gna14/atp2b3b/mapk12a/adcy9/ppp2r5b 
 
 
 dre00020 
  dre00020  
 Citrate cycle (TCA cycle) 
 0.0115090 
 12 
 suclg1/mdh1aa/sdha/dlat/dldh/idh1/pcxb/pdhb/ogdha/aclya/aco2/pck2 
 
 
 dre00830 
  dre00830  
 Retinol metabolism 
 0.0147549 
 13 
 adh5/cyp3a65/ugt1ab/ugt1a1/aldh1a2/bco1/lratb.1/dhrs3b/si:ch1073-13h15.3/cyp26a1/lrata/dgat1a/aox5 
 
 
 dre01200 
  dre01200  
 Carbon metabolism 
 0.0254048 
 29 
 aldh6a1/suclg1/mdh1aa/gldc/adh5/fbp1b/shmt2/sdha/cat/dlat/acads/dldh/idh1/acss1/aldob/acox1/acss2/pkma/pcxb/pdhb/got1/ogdha/h6pd/aco2/gck/hk1/pfkpb/g6pd/hkdc1 
 
 
 dre04912 
  dre04912  
 GnRH signaling pathway 
 0.0476419 
 27 
 camk2b/cacna1fb/camk2a/camk2d2/camk2g1/grb2b/gna11b/camk2g2/lhb/cacna1da/gnaq/camk2d1/mapk3/mapk1/egr1/cacna1sa/itpr3/elk1/camk2b1/pld2/gnrh2/ptk2bb/prkacab/hrasb/gna14/mapk12a/adcy9 
 
 
 
 
 
  GO Analysis  
 
  [MF] Over Representation Analysis  
 The ORA method takes a group of significative DEGs (only DEGs, upregulated DEGs or downregulated DEGs) and performs a hypergeometric test for each term of the selected functional category. 
  Barplot  
 The plot shows the functional top significative terms in ascendant order by adjusted pvalue. The color represents the associated adjusted pvalue. The X axis represents the proportion of the known genes for a given functional term that are identified in the expression data. 
 
   
 
  Dotplot  
 The plot shows the top functional terms in descendant order by gene ratio. This ratio is the proportion of the known genes for a given functional term that are identified in the expression data. The color represents the associated adjusted pvalue. The X axis represents the gene ratio and the dot size the number of DEG genes associated to the functional term. 
 
   
 
  Gene-Concept Network  
 The network connects the top functional terms (brown nodes) to their associated genes (grey or colored nodes). The size of the functional terms shows the number of connected genes. 
 
   
 
  Enrich Map plot  
 The network connects the top functional terms (nodes) between them trought their associates genes (grey edges, thickness represents the number of shared genes.). The size of the functional terms shows the number of connected genes and the color the adjusted pvalue of the functional term. 
 
   
 
  Heatplot  
 Top functional terms are show in the Y axis whereas in the X axis are show the associated genes to them. The coloured squares indicates the fold change value. 
 
   
 
  Upsetplot  
 Top functional terms are listed the Y axis with the number of genes associated to them (Set size). Each column of dots marks one cluster of functional terms. The X axis indicates how many genes are shared exclusively for the members of one cluster. 
 
   
 
 
 
  [BP] Over Representation Analysis  
 The ORA method takes a group of significative DEGs (only DEGs, upregulated DEGs or downregulated DEGs) and performs a hypergeometric test for each term of the selected functional category. 
  Barplot  
 The plot shows the functional top significative terms in ascendant order by adjusted pvalue. The color represents the associated adjusted pvalue. The X axis represents the proportion of the known genes for a given functional term that are identified in the expression data. 
 
   
 
  Dotplot  
 The plot shows the top functional terms in descendant order by gene ratio. This ratio is the proportion of the known genes for a given functional term that are identified in the expression data. The color represents the associated adjusted pvalue. The X axis represents the gene ratio and the dot size the number of DEG genes associated to the functional term. 
 
   
 
  Gene-Concept Network  
 The network connects the top functional terms (brown nodes) to their associated genes (grey or colored nodes). The size of the functional terms shows the number of connected genes. 
 
   
 
  Enrich Map plot  
 The network connects the top functional terms (nodes) between them trought their associates genes (grey edges, thickness represents the number of shared genes.). The size of the functional terms shows the number of connected genes and the color the adjusted pvalue of the functional term. 
 
   
 
  Heatplot  
 Top functional terms are show in the Y axis whereas in the X axis are show the associated genes to them. The coloured squares indicates the fold change value. 
 
   
 
  Upsetplot  
 Top functional terms are listed the Y axis with the number of genes associated to them (Set size). Each column of dots marks one cluster of functional terms. The X axis indicates how many genes are shared exclusively for the members of one cluster. 
 
   
 
 
 
  [CC] Over Representation Analysis  
 The ORA method takes a group of significative DEGs (only DEGs, upregulated DEGs or downregulated DEGs) and performs a hypergeometric test for each term of the selected functional category. 
  Barplot  
 The plot shows the functional top significative terms in ascendant order by adjusted pvalue. The color represents the associated adjusted pvalue. The X axis represents the proportion of the known genes for a given functional term that are identified in the expression data. 
 
   
 
  Dotplot  
 The plot shows the top functional terms in descendant order by gene ratio. This ratio is the proportion of the known genes for a given functional term that are identified in the expression data. The color represents the associated adjusted pvalue. The X axis represents the gene ratio and the dot size the number of DEG genes associated to the functional term. 
 
   
 
  Gene-Concept Network  
 The network connects the top functional terms (brown nodes) to their associated genes (grey or colored nodes). The size of the functional terms shows the number of connected genes. 
 
   
 
  Enrich Map plot  
 The network connects the top functional terms (nodes) between them trought their associates genes (grey edges, thickness represents the number of shared genes.). The size of the functional terms shows the number of connected genes and the color the adjusted pvalue of the functional term. 
 
   
 
  Heatplot  
 Top functional terms are show in the Y axis whereas in the X axis are show the associated genes to them. The coloured squares indicates the fold change value. 
 
   
 
  Upsetplot  
 Top functional terms are listed the Y axis with the number of genes associated to them (Set size). Each column of dots marks one cluster of functional terms. The X axis indicates how many genes are shared exclusively for the members of one cluster. 
 
   
 
 
 
  [All] Over Representation Analysis Unified  
 This category will aggregate the results for all the selected GO ontologies 
  Barplot  
 The plot shows the functional top significative terms in ascendant order by adjusted pvalue. The color represents the associated adjusted pvalue. The X axis represents the proportion of the known genes for a given functional term that are identified in the expression data. 
 
   
 
  Dotplot  
 The plot shows the top functional terms in descendant order by gene ratio. This ratio is the proportion of the known genes for a given functional term that are identified in the expression data. The color represents the associated adjusted pvalue. The X axis represents the gene ratio and the dot size the number of DEG genes associated to the functional term. 
 
   
 
  Gene-Concept Network  
 The network connects the top functional terms (brown nodes) to their associated genes (grey or colored nodes). The size of the functional terms shows the number of connected genes. 
 
   
 
  Enrich Map plot  
 The network connects the top functional terms (nodes) between them trought their associates genes (grey edges, thickness represents the number of shared genes.). The size of the functional terms shows the number of connected genes and the color the adjusted pvalue of the functional term. 
 
   
 
  Heatplot  
 Top functional terms are show in the Y axis whereas in the X axis are show the associated genes to them. The coloured squares indicates the fold change value. 
 
   
 
  Upsetplot  
 Top functional terms are listed the Y axis with the number of genes associated to them (Set size). Each column of dots marks one cluster of functional terms. The X axis indicates how many genes are shared exclusively for the members of one cluster. 
 
   
 
 
 
 
  REACTOME analysis  
 
  Over Representation Analysis  
 The ORA method takes a group of significative DEGs (only DEGs, upregulated DEGs or downregulated DEGs) and performs a hypergeometric test for each term of the selected functional category. 
  Barplot  
 The plot shows the functional top significative terms in ascendant order by adjusted pvalue. The color represents the associated adjusted pvalue. The X axis represents the proportion of the known genes for a given functional term that are identified in the expression data. 
 
   
 
  Dotplot  
 The plot shows the top functional terms in descendant order by gene ratio. This ratio is the proportion of the known genes for a given functional term that are identified in the expression data. The color represents the associated adjusted pvalue. The X axis represents the gene ratio and the dot size the number of DEG genes associated to the functional term. 
 
   
 
  Gene-Concept Network  
 The network connects the top functional terms (brown nodes) to their associated genes (grey or colored nodes). The size of the functional terms shows the number of connected genes. 
 
   
 
  Enrich Map plot  
 The network connects the top functional terms (nodes) between them trought their associates genes (grey edges, thickness represents the number of shared genes.). The size of the functional terms shows the number of connected genes and the color the adjusted pvalue of the functional term. 
 
   
 
  Heatplot  
 Top functional terms are show in the Y axis whereas in the X axis are show the associated genes to them. The coloured squares indicates the fold change value. 
 
   
 
  Upsetplot  
 Top functional terms are listed the Y axis with the number of genes associated to them (Set size). Each column of dots marks one cluster of functional terms. The X axis indicates how many genes are shared exclusively for the members of one cluster. 
 
   
 
 
 
 


 
 

 

 

 
 

 
 
