# Supplementary material for: An improved de novo assembling and polishing of Solea senegalensis transcriptome shed light on retinoic acid signalling in larvae
Source: Sci Rep. 2020 Nov 26;10:20654. doi: 10.1038/s41598-020-77201-z (PMC7691524; doi:10.1038/s41598-020-77201-z)
Supplement: Supplementary file 2 — Supplementary file 4. [file 41598_2020_77201_MOESM2_ESM.zip › FA_Common-Specific/DEAB_24h_specific.html]

/mnt/home/users/bio\_267\_uma/josecordoba/proyectos/lenguado/RA\_DE/RA\_all\_comparisons/v5\_transcriptome/DEAB\_24h\_specific/DEAB\_24h\_specific/functional\_report.utf8.md


# **DEgenes Hunter - Functional analysis report**

## **Used data in this analysis**

Specifically, in this experiment set, known experiment labels are:

- EXPERIMENT NAMES NOT AVAILABLE

## **General description**

This report contains all the functional information that was requested by the options when functional\_Hunter.R was executed. The functional categories can be:

- KEGG pathways
- GO:
  - Biological Process
  - Molecular Function
  - Cellular Component
- Reactome pathways
- Custom nomenclature

All the functional categories are computed with CluterProfiler and GO caterogires are computed also with TopGo. Some sections will not show if there are not sinficative results. Each category is analysed using Over representation analysis (ORA) and Gene Set Analysis (GSEA). The ORA method takes a group of significative DEGs (only DEGs, upregulated DEGs or downregulated DEGs) and performs a hypergeometric test for each term of the selected functional category. In the case of the GSEA method, all the genes are sorted by their fold-change and the algorithm scan which genes with similar fold-change shares a term of the selected functional category.

Statistics about input results obtained from DEGenes Expression Hunter are:

| Var1 | Freq |
| --- | --- |
| FILTERED\_OUT | 20351 |
| NOT\_DEG | 27039 |
| POSSIBLE\_DEG | 1995 |
| PREVALENT\_DEG | 1258 |

## **KEGG analysis**

### **Over Representation Analysis**

The ORA method takes a group of significative DEGs (only DEGs, upregulated DEGs or downregulated DEGs) and performs a hypergeometric test for each term of the selected functional category.

**Barplot**

The plot shows the functional top significative terms in ascendant order by adjusted pvalue. The color represents the associated adjusted pvalue. The X axis represents the proportion of the known genes for a given functional term that are identified in the expression data.

**Dotplot**

The plot shows the top functional terms in descendant order by gene ratio. This ratio is the proportion of the known genes for a given functional term that are identified in the expression data. The color represents the associated adjusted pvalue. The X axis represents the gene ratio and the dot size the number of DEG genes associated to the functional term.

**Gene-Concept Network**

The network connects the top functional terms (brown nodes) to their associated genes (grey or colored nodes). The size of the functional terms shows the number of connected genes.

**Enrich Map plot**

The network connects the top functional terms (nodes) between them trought their associates genes (grey edges, thickness represents the number of shared genes.). The size of the functional terms shows the number of connected genes and the color the adjusted pvalue of the functional term.

**Heatplot**

Top functional terms are show in the Y axis whereas in the X axis are show the associated genes to them. The coloured squares indicates the fold change value.

**Upsetplot**

Top functional terms are listed the Y axis with the number of genes associated to them (Set size). Each column of dots marks one cluster of functional terms. The X axis indicates how many genes are shared exclusively for the members of one cluster.

## **ClusterProfiler KEGG table**

Detailed table with the data obtained from KEGG pathways (ORA method only). For each pathway, the identifier, link to KEGG DB with the detected genes, name, adjusted pvalue and genes (count and ids)

|  | ID | Description | p.adjust | Count | geneID |
| --- | --- | --- | --- | --- | --- |
| dre05168 | dre05168 | Herpes simplex virus 1 infection | 0.0389096 | 19 | mhc2b/pdia3/si:busm1-48c11.3/ifih1/b2m/stat1a/irf9/tapbp.2/tapbp.1/tap2a/tmem173/casp3a/cd74a/tbk1/irf7/pkz/tlr3/eif2ak2/b2ml |
| dre03050 | dre03050 | Proteasome | 0.0389096 | 9 | psmb10/psmb9a/psma6l/psme1/psme2/psmb13a/psmb12/psmb8a/psmf1 |

## **GO Analysis**

### **[MF] Over Representation Analysis**

The ORA method takes a group of significative DEGs (only DEGs, upregulated DEGs or downregulated DEGs) and performs a hypergeometric test for each term of the selected functional category.

**Barplot**

The plot shows the functional top significative terms in ascendant order by adjusted pvalue. The color represents the associated adjusted pvalue. The X axis represents the proportion of the known genes for a given functional term that are identified in the expression data.

**Dotplot**

The plot shows the top functional terms in descendant order by gene ratio. This ratio is the proportion of the known genes for a given functional term that are identified in the expression data. The color represents the associated adjusted pvalue. The X axis represents the gene ratio and the dot size the number of DEG genes associated to the functional term.

**Gene-Concept Network**

The network connects the top functional terms (brown nodes) to their associated genes (grey or colored nodes). The size of the functional terms shows the number of connected genes.

**Enrich Map plot**

The network connects the top functional terms (nodes) between them trought their associates genes (grey edges, thickness represents the number of shared genes.). The size of the functional terms shows the number of connected genes and the color the adjusted pvalue of the functional term.

**Heatplot**

Top functional terms are show in the Y axis whereas in the X axis are show the associated genes to them. The coloured squares indicates the fold change value.

**Upsetplot**

Top functional terms are listed the Y axis with the number of genes associated to them (Set size). Each column of dots marks one cluster of functional terms. The X axis indicates how many genes are shared exclusively for the members of one cluster.

### **[BP] Over Representation Analysis**

The ORA method takes a group of significative DEGs (only DEGs, upregulated DEGs or downregulated DEGs) and performs a hypergeometric test for each term of the selected functional category.

**Barplot**

The plot shows the functional top significative terms in ascendant order by adjusted pvalue. The color represents the associated adjusted pvalue. The X axis represents the proportion of the known genes for a given functional term that are identified in the expression data.

**Dotplot**

The plot shows the top functional terms in descendant order by gene ratio. This ratio is the proportion of the known genes for a given functional term that are identified in the expression data. The color represents the associated adjusted pvalue. The X axis represents the gene ratio and the dot size the number of DEG genes associated to the functional term.

**Gene-Concept Network**

The network connects the top functional terms (brown nodes) to their associated genes (grey or colored nodes). The size of the functional terms shows the number of connected genes.

**Enrich Map plot**

The network connects the top functional terms (nodes) between them trought their associates genes (grey edges, thickness represents the number of shared genes.). The size of the functional terms shows the number of connected genes and the color the adjusted pvalue of the functional term.

**Heatplot**

Top functional terms are show in the Y axis whereas in the X axis are show the associated genes to them. The coloured squares indicates the fold change value.

**Upsetplot**

Top functional terms are listed the Y axis with the number of genes associated to them (Set size). Each column of dots marks one cluster of functional terms. The X axis indicates how many genes are shared exclusively for the members of one cluster.

### **[CC] Over Representation Analysis**

The ORA method takes a group of significative DEGs (only DEGs, upregulated DEGs or downregulated DEGs) and performs a hypergeometric test for each term of the selected functional category.

**Barplot**

The plot shows the functional top significative terms in ascendant order by adjusted pvalue. The color represents the associated adjusted pvalue. The X axis represents the proportion of the known genes for a given functional term that are identified in the expression data.

**Dotplot**

The plot shows the top functional terms in descendant order by gene ratio. This ratio is the proportion of the known genes for a given functional term that are identified in the expression data. The color represents the associated adjusted pvalue. The X axis represents the gene ratio and the dot size the number of DEG genes associated to the functional term.

**Gene-Concept Network**

The network connects the top functional terms (brown nodes) to their associated genes (grey or colored nodes). The size of the functional terms shows the number of connected genes.

**Enrich Map plot**

The network connects the top functional terms (nodes) between them trought their associates genes (grey edges, thickness represents the number of shared genes.). The size of the functional terms shows the number of connected genes and the color the adjusted pvalue of the functional term.

**Heatplot**

Top functional terms are show in the Y axis whereas in the X axis are show the associated genes to them. The coloured squares indicates the fold change value.

**Upsetplot**

Top functional terms are listed the Y axis with the number of genes associated to them (Set size). Each column of dots marks one cluster of functional terms. The X axis indicates how many genes are shared exclusively for the members of one cluster.

### **[All] Over Representation Analysis Unified**

This category will aggregate the results for all the selected GO ontologies

**Barplot**

The plot shows the functional top significative terms in ascendant order by adjusted pvalue. The color represents the associated adjusted pvalue. The X axis represents the proportion of the known genes for a given functional term that are identified in the expression data.

**Dotplot**

The plot shows the top functional terms in descendant order by gene ratio. This ratio is the proportion of the known genes for a given functional term that are identified in the expression data. The color represents the associated adjusted pvalue. The X axis represents the gene ratio and the dot size the number of DEG genes associated to the functional term.

**Gene-Concept Network**

The network connects the top functional terms (brown nodes) to their associated genes (grey or colored nodes). The size of the functional terms shows the number of connected genes.

**Enrich Map plot**

The network connects the top functional terms (nodes) between them trought their associates genes (grey edges, thickness represents the number of shared genes.). The size of the functional terms shows the number of connected genes and the color the adjusted pvalue of the functional term.

**Heatplot**

Top functional terms are show in the Y axis whereas in the X axis are show the associated genes to them. The coloured squares indicates the fold change value.

**Upsetplot**

Top functional terms are listed the Y axis with the number of genes associated to them (Set size). Each column of dots marks one cluster of functional terms. The X axis indicates how many genes are shared exclusively for the members of one cluster.

## **REACTOME analysis**

### **Over Representation Analysis**

The ORA method takes a group of significative DEGs (only DEGs, upregulated DEGs or downregulated DEGs) and performs a hypergeometric test for each term of the selected functional category.

**Barplot**

The plot shows the functional top significative terms in ascendant order by adjusted pvalue. The color represents the associated adjusted pvalue. The X axis represents the proportion of the known genes for a given functional term that are identified in the expression data.

**Dotplot**

The plot shows the top functional terms in descendant order by gene ratio. This ratio is the proportion of the known genes for a given functional term that are identified in the expression data. The color represents the associated adjusted pvalue. The X axis represents the gene ratio and the dot size the number of DEG genes associated to the functional term.

**Gene-Concept Network**

The network connects the top functional terms (brown nodes) to their associated genes (grey or colored nodes). The size of the functional terms shows the number of connected genes.

**Enrich Map plot**

The network connects the top functional terms (nodes) between them trought their associates genes (grey edges, thickness represents the number of shared genes.). The size of the functional terms shows the number of connected genes and the color the adjusted pvalue of the functional term.

**Heatplot**

Top functional terms are show in the Y axis whereas in the X axis are show the associated genes to them. The coloured squares indicates the fold change value.

**Upsetplot**

Top functional terms are listed the Y axis with the number of genes associated to them (Set size). Each column of dots marks one cluster of functional terms. The X axis indicates how many genes are shared exclusively for the members of one cluster.
