# Supplementary material for: An improved de novo assembling and polishing of Solea senegalensis transcriptome shed light on retinoic acid signalling in larvae
Source: Sci Rep. 2020 Nov 26;10:20654. doi: 10.1038/s41598-020-77201-z (PMC7691524; doi:10.1038/s41598-020-77201-z)
Supplement: Supplementary file 3 — Supplementary file 5. [file 41598_2020_77201_MOESM3_ESM.zip › CTRL_vs_TTNPB_24/DEG_report.html]

main\_report.utf8


# **DEgenes Hunter - Differential expression analysis report**

## **Details of the input data**

### **First group of samples (to be referred to as control in the rest of the report)**

| Sample Names: |
| --- |
| ctrl\_24h\_1 |
| ctrl\_24h\_2 |
| ctrl\_24h\_3 |

### **Second group of samples (to be referred to as treatment in the rest of the report)**

| Sample Names: |
| --- |
| trt\_TTNPB\_24h\_1 |
| trt\_TTNPB\_24h\_2 |
| trt\_TTNPB\_24h\_3 |

Note: A positive log fold change shows higher expression in the treatment group; a negative log fold change represents higher expression in the control group.

## **Data quality control (QC)**

### **Correlation between samples:**

Here we show scatterplots comparing expression levels for all genes between the different samples, for i) all controls, ii) all treatment samples and iii) for all samples together.

These plots will only be produced when the total number of samples to compare within a group is less than or equal to 10.

### **Correlation between control samples:**

Replicates within the same group tend to have Pearson correlation coefficients >= 0.96. Lower values may indicate problems with the samples.

### **Correlation between treatment samples:**

Replicates within the same group tend to have Pearson correlation coefficients >= 0.96. Lower values may indicate problems with the samples.

### **Correlation between samples: All vs all replicates**

Correlation coefficients tend to be slightly higher between replicates from the same group than between replicates from different groups. If this is not the case, it may indicate mislabelling or other potential issues.

### **Heatmap and clustering showing correlation between replicates**

**BROWN: higher correlation; YELLOW: lower**

### **Principal Component Analysis**

This is a PCA plot of the count values following rlog normalization from the DESeq2 package:

The samples are shown in the 2D plane and distributed by their first two principal components. This type of plot is useful for visualizing the overall effect of experimental covariates and batch effects. It is also useful for identifying outlier samples. Control and treatment samples respectively may cluster together.

### **Visualizing normalization results**

These boxplots show the distributions of count data before and after normalization (shown for normalization method DESeq2):

Before normalization:

After normalization:

### **Samples differences by all counts normalized**:

All counts were normalizated by DESeq2 algorithm. This count were scaled by log10 and plotted in a heatmap.

```
## 
## 
## This plot can not be generated because more than 50 000 genes have passed the filters and it can cause memory problems.
```

## **DEgenes Hunter results**

### **Gene classification by DEgenes Hunter**

DEgenes Hunter uses multiple DE detection packages to analyse all genes in the input count table and labels them accordingly:

- **Filtered out:** Genes discarded during the filtering process as showing no or very low expression.
- **Prevalent DEG:** Genes considered as differentially expressed (DE) by at least 4 packages, as specified by the `minpack_common` argument.
- **Possible DEG:** Genes considered DE by at least one of the DE detection packages.
- **Not DEG:** Genes not considered DE in any package.

This barplot shows the total number of genes passing each stage of analysis - from the total number of genes in the input table of counts, to the genes surviving the expression filter, to the genes detected as DE by one package, to the genes detected by at least 4 packages.

### **Package DEG detection stats**

This is the Venn Diagram of all possible DE genes (DEGs) according to at least on of the DE detection packages employed:

### **FDR gene-wise benchmarking**

Benchmark of false positive calling (Image extracted from {“padj\_prevalent\_DEGs.pdf”} file):

Boxplot of FDR values among all genes with an FDR <= 0.05 in at least one DE detection package

The complete results of the DEgenes Hunter differential expression analysis can be found in the “hunter\_results\_table.txt” file in the Common\_results folder

## **DE detection package specific results**

Various plots specific to each package are shown below:

### **DESeq2 normalization effects**:

This plot compares the effective library size with raw library size

```
## `geom_smooth()` using formula 'y ~ x'
```

The effective library size is the factor used by DESeq2 normalizatioin algorithm for eahc sample. The effective library size must be dependent of raw library size.

### **DESeq2 MA plot**:

This is the MA plot from DESeq2 package:

In DESeq2, the MA-plot (log ratio versus abundance) shows the log2 fold changes are attributable to a given variable over the mean of normalized counts. Points will be colored red if the adjusted Pvalue is less than 0.1. Points which fall out of the window are plotted as open triangles pointing either up or down.

A table containing the DESeq2 DEGs is provided: in Results\_DESeq2/DEgenes\_DESEq2.txt

A table containing the DESeq2 normalized counts is provided in Results\_DESeq2/Normalized\_counts\_DESEq2.txt

### **Differences between samples by PREVALENT DEGs normalized counts**:

Counts of prevalent DEGs were normalizated by DESeq2 algorithm. This count were scaled by log10 and plotted in a heatmap.

### **edgeR MA plot**

This is the MA plot from edgeR package:

Differential gene expression data can be visualized as MA-plots (log ratio versus abundance) where each dot represents a gene. The differentially expressed genes are colored red and the non-differentially expressed ones are colored black.

A table containing the edgeR DEGs is provided in Results\_edgeR/DEgenes\_edgeR.txt

A table containing the edgeR normalized counts is provided in Results\_edgeR/Normalized\_counts\_edgeR.txt

### **limma Volcano plot**

Volcano plot of log2-fold change versus -log10 of adjusted p-values for all genes according to the analysis with limma:

A table containing the limma DEGs is provided in Results\_limma/DEgenes\_limma.txtA table containing the limma normalized counts is provided in Results\_limma/Normalized\_counts\_limma.txt

### **NOISeq Expressionplot**

This is the summary plot for (M,D) values (black) and the differentially expressed genes (red) from the NOISeq package (Image extracted from {‘ExpressionPlot.pdf’} file):

A table containing the NOISeq DEGs is provided in Results\_NOISeq/DEgenes\_NOISeq.txt.

A table containing the NOISeq normalized counts is provided in Results\_NOISeq/Normalized\_counts\_NOISeq.txt

## **Detailed package results comparation**

This is an advanced section in order to compare the output of the packages used to perform data analysis. The data shown here does not necessarilly have any biological implication.

### **P-value Distributions**

Distributions of p-values, unadjusted and adjusted for multiple testing (FDR)

### **FDR Correlations**

Correlations of adjusted p-values, adjusted for multiple testing (FDR) and for log Fold Change.

### **Values of options used to run DEGenesHunter**

First column contains the option names; second column contains the given values for each option in this run.

|  | opt\_orig |
| --- | --- |
| input\_file | /mnt/scratch/users/bio\_267\_uma/josecordoba/leng/transcriptoma/DE\_retinoic\_v4/DEGenesHunter\_results/CTL\_vs\_TTNPB\_24/final\_counts.txt |
| reads | 2 |
| minlibraries | 2 |
| filter\_type | separate |
| output\_files | /mnt/scratch/users/bio\_267\_uma/josecordoba/leng/transcriptoma/DE\_retinoic\_v4/DEGenesHunter\_results/CTL\_vs\_TTNPB\_24 |
| p\_val\_cutoff | 0.05 |
| lfc | 1 |
| modules | DELN |
| minpack\_common | 4 |
| target\_file | /mnt/home/users/bio\_267\_uma/josecordoba/proyectos/lenguado/ensamblaje\_transcriptoma/DE\_retinoic/DEG\_workflow\_v4/TARGETS/CTL\_vs\_TTNPB\_24\_target.txt |
| model\_variables |  |
| numerics\_as\_factors | TRUE |
| custom\_model | FALSE |
| string\_factors | drug,time |
| numeric\_factors |  |
| WGCNA\_memory | 5000 |
| WGCNA\_deepsplit | 2 |
| WGCNA\_min\_genes\_cluster | 15 |
| WGCNA\_detectcutHeight | 0.995 |
| WGCNA\_mergecutHeight | 0.15 |
| WGCNA\_all | FALSE |
| WGCNA\_blockwiseNetworkType | signed |
| WGCNA\_blockwiseTOMType | signed |
| debug | FALSE |
| Debug | /mnt/scratch/users/bio\_267\_uma/josecordoba/leng/transcriptoma/DE\_retinoic\_v4/DEGenesHunter\_results/CTL\_vs\_TTNPB\_24/debug\_files/DH\_debug\_session.RData |
| help | FALSE |
